# ChemoNETosis in Cancer: A Comprehensive Review of Treatment-Induced NET Formation and Therapeutic Consequences

**DOI:** 10.3390/cells15060536

**Published:** 2026-03-17

**Authors:** Bojan Stojanovic, Bojana S. Stojanovic, Milica Dimitrijevic Stojanovic, Aleksandar Cvetkovic, Bojan Milosevic, Vesna Vulovic, Ivana Milivojcevic Bevc, Andra Jevtovic, Danijela Tasic-Uros, Sanja Knezevic, Aleksandar Matic, Marina Markovic, Katarina Milojevic, Verica Vukicevic, Danijela Bazic Sretenovic, Sladjan Petrovic, Tatjana Boskovic Matic, Milos Zivic, Tatjana Lazarevic

**Affiliations:** 1 Department of Surgery, Faculty of Medical Sciences, University of Kragujevac, 34000 Kragujevac, Serbia; bojan.stojanovic01@gmail.com (B.S.);; 2 Center for Molecular Medicine and Stem Cell Research, Faculty of Medical Sciences, University of Kragujevac, 34000 Kragujevac, Serbia; 3 Department of Pathophysiology, Faculty of Medical Sciences, University of Kragujevac, 34000 Kragujevac, Serbia; 4 Department of Pathology, Faculty of Medical Sciences, University of Kragujevac, 34000 Kragujevac, Serbia; 5 Department of Surgery, University Hospital Medical Center Bežanijska Kosa, 11000 Belgrade, Serbia; 6 City Medical Emergency Department, 11000 Belgrade, Serbia; 7 Department of Otorhinolaryngology, Faculty of Medical Sciences, University of Kragujevac, 34000 Kragujevac, Serbia; 8 Department of Pediatrics, Faculty of Medical Sciences, University of Kragujevac, 34000 Kragujevac, Serbia; 9 Department of Internal Medicine, Faculty of Medical Sciences, University of Kragujevac, 34000 Kragujevac, Serbia; 10 Department of Medical Oncology, University Clinical Center Kragujevac, 34000 Kragujevac, Serbia; 11 Department of Surgery, Health Center Prokuplje, 18400 Prokuplje, Serbia; 12 Department of Neurology, Faculty of Medical Sciences, University of Kragujevac, 34000 Kragujevac, Serbia; 13 Department of Dentistry, Faculty of Medical Sciences, University of Kragujevac, 34000 Kragujevac, Serbia

**Keywords:** ChemoNETosis, NETosis, neutrophil extracellular traps, chemoresistance, tumor microenvironment

## Abstract

**Highlights:**

**What are the main findings?**

- ChemoNETosis represents a treatment-induced neutrophil extracellular trap (NET) program in which cytotoxic chemotherapy alters the cytokine and chemokine landscape of the tumor microenvironment, including signals such as interleukin 1 beta (IL 1β) and the C X C motif chemokine ligand 1 and 5–C X C chemokine receptor 2 (CXCL1 and CXCL5–CXCR2) axis. These changes facilitate neutrophil recruitment and activation, culminating in NET deposition within primary tumors and metastatic niches.
- NET-enriched microenvironments can contribute mechanistically to chemoresistance by promoting epithelial to mesenchymal transition and tumor cell plasticity, for example through NET scaffold-associated activation of latent transforming growth factor beta (TGF β) signaling. In selected clinical contexts, this process may also connect therapy-induced inflammatory stress with vascular dysfunction and systemic toxicities.

**What are the implications of the main findings?**

- ChemoNETosis identifies therapeutically actionable checkpoints that may be incorporated into rational combination strategies. These include enzymatic NET disruption using deoxyribonuclease (DNase), inhibition of peptidylarginine deiminase 4 (PAD4) to prevent chromatin decondensation, and targeting upstream inflammatory circuits such as IL 1β or CXCR2 signaling, with the aim of restoring chemotherapy and potentially immunotherapy sensitivity without broadly compromising host defense.
- Circulating and tissue-based NET biomarkers, including citrullinated histone H3 (CitH3), neutrophil elastase–DNA (NE–DNA) complexes, and cell-free DNA (cfDNA), may assist in patient stratification and treatment monitoring, and could help identify resistance phenotypes in which NET-directed adjunctive strategies are most likely to provide clinical benefit.

**Abstract:**

ChemoNETosis represents a distinct form of therapy-induced innate immune activation, in which cytotoxic chemotherapy alters the tumor microenvironment (TME) in ways that attract and stimulate neutrophils, ultimately triggering the release of neutrophil extracellular traps (NETs). Unlike classical NETosis, which typically arises in response to infection or sterile inflammation, chemoNETosis is initiated by treatment-related danger signals and chemokine–cytokine loops that reshape the immune landscape and promote the formation of NET-rich metastatic niches. These NET structures serve not only as physical scaffolds but also as bioactive platforms enriched with proteases, reactive oxygen species, and enzymes capable of activating growth factors, collectively driving epithelial–mesenchymal transition, enhanced tumor cell plasticity, immune cell exclusion, changes in vascular permeability, and the development of chemotherapy resistance. While predominantly associated with tumor-promoting effects, chemoNETosis may, under specific genetic or metabolic conditions, contribute to antitumor responses, reflecting its context-dependent plasticity. In this review, we present what is, to our knowledge, the first in-depth synthesis of chemoNETosis across solid tumors, with a focus on key mechanistic nodes and translational perspectives.

## 1. Introduction

ChemoNETosis refers to a form of therapy-induced inflammation in which cytotoxic chemotherapy, beyond its direct cytotoxicity toward malignant cells, alters the cytokine and chemokine balance within the TME in a manner that promotes neutrophil recruitment and activation. This inflammatory cascade leads to the release of NETs, web-like structures composed of DNA and granule proteins, which have emerged as key contributors to reduced chemotherapy efficacy and the development of clinically significant resistance. Within this context, chemotherapy-induced NETosis represents a biologically dynamic interface between tumor-derived stress responses and host innate immunity. Once released, NETs create a structural and enzymatic scaffold that enhances tumor cell plasticity, supports survival under drug pressure, and facilitates the establishment of metastatic niches. Preclinical models have shown that disruption of this pathway—through inhibition of PAD4 or enzymatic degradation of NETs using DNase—can partially restore treatment responsiveness, suggesting that chemoNETosis represents a modifiable component of resistance biology. This review offers, to our knowledge, the first dedicated synthesis of chemoNETosis across solid tumors, with a focus on its mechanistic underpinnings and disease-specific relevance in the context of treatment resistance.

## 2. Chemoresistance as a Multiscale Problem: Tumor-Intrinsic Adaptation and Microenvironmental Protection

Cancer remains one of the leading public health challenges globally, accounting for nearly one in six deaths worldwide. According to GLOBOCAN 2022 estimates, approximately 20 million new cancer cases were diagnosed in that year (around 18.74 million when excluding nonmelanoma skin cancers), with nearly 9.7 million cancer-related deaths, highlighting the persistent and growing burden of this disease across populations [1]. Modern cancer care is increasingly personalized, multidisciplinary, and adapted to disease stage. It typically involves a combination of local treatments such as surgery and radiotherapy, and systemic therapies including cytotoxic chemotherapy, hormone therapies, targeted drugs, and immunotherapies [2].

The development of metastases remains the primary cause of cancer-related death [3]. For many patients with advanced-stage solid tumors or hematologic malignancies, systemic cytotoxic chemotherapy continues to serve as a central component of treatment [4]. Yet, once cancer spreads beyond the primary site, therapeutic success becomes more elusive. The challenge lies in managing a heterogeneous population of metastatic lesions that differ in vascularization, immune landscape, proliferative behavior, and metabolic profile [5,6,7]. Under the continuous pressure of cytotoxic agents, resistant malignant subclones with survival advantages may gradually dominate [5]. At the same time, chemotherapy-induced cellular stress can promote greater phenotypic adaptability, enabling cancer cells to persist in adverse environments and eventually drive disease progression [8,9]. In this context, the clinical problem extends beyond incomplete tumor eradication. It involves the swift evolution of tumor populations that respond progressively less to treatment, which is a critical factor contributing to poor outcomes in metastatic disease despite the availability of multiple therapeutic classes.

Chemoresistance refers to a decrease or complete loss of tumor responsiveness to antineoplastic agents, which may be present from the outset (intrinsic resistance) or develop following treatment exposure (acquired resistance) [10]. At the mechanistic level, resistance arises from both intrinsic cellular adaptations and external protection offered by TME [11]. Tumor cell-intrinsic mechanisms include enhanced activity of efflux transporters that reduce intracellular drug accumulation, improved DNA repair capacity, evasion of apoptosis through altered balance of B-cell lymphoma 2 (BCL-2) family proteins and disrupted tumor protein p53 (TP53) signaling, and activation of survival pathways such as those regulated by phosphoinositide 3-kinase/protein kinase B (PI3K/AKT), mitogen-activated protein kinase (MAPK), and nuclear factor kappa B (NF-κB) [12,13,14,15]. These changes are frequently accompanied by epigenetic reprogramming and lineage plasticity [16]. Simultaneously, the TME—composed of abnormal vasculature, cancer-associated fibroblasts, immune cell subsets, and extracellular matrix—generates uneven drug distribution, secretes prosurvival cytokines and metabolites, and induces hypoxic and acidic conditions that further reduce drug efficacy [17,18]. The interplay between cellular and microenvironmental resistance not only compromises therapeutic response but also fosters metastatic progression, forming a unified clinical challenge that underpins treatment failure in advanced cancer.

## 3. Neutrophils in Cancer: Recruitment, Reprogramming, and Consequences for Therapy

Neutrophils, also known as polymorphonuclear leukocytes (PMNs), are fully differentiated myeloid cells produced in the bone marrow and constitute the most abundant type of circulating leukocytes in humans [19]. Due to their short lifespan in peripheral blood, maintenance of baseline immune surveillance relies on continuous production in the marrow and rapid deployment in response to tissue damage or infection [20]. As essential components of the innate immune system, neutrophils swiftly detect inflammatory signals, migrate to affected sites, and execute microbial clearance through multiple coordinated strategies [21]. These include phagocytosis, release of proteolytic and antimicrobial enzymes via degranulation, and the generation of reactive oxygen species (ROS) [21]. In recent years, it has become increasingly evident that the role of neutrophils extends beyond classical host defense. They are actively recruited to tumor microenvironments and metastatic niches by chemokines and damage-associated molecular patterns (DAMPs), where they participate in reshaping the local tissue context [22,23]. Through secretion of proinflammatory mediators, proangiogenic and growth-promoting factors, and modulators of immune activity, neutrophils influence a range of tumor-associated processes, including cell survival, invasiveness, neovascularization, and the efficacy of antitumor immune responses [24]. These effects are often context-dependent, positioning neutrophils as dynamic contributors to both tumor progression and metastatic spread.

### 3.1. Tumor-Associated Neutrophils in Cancer Biology

Tumor-associated neutrophils (TANs) represent a specialized subset of neutrophils that infiltrate the TME, where they undergo functional reprogramming driven by local inflammatory, metabolic, and cellular signals [25]. Unlike circulating neutrophils, TANs adopt distinct transcriptional and phenotypic profiles that reflect altered patterns of migration, prolonged survival, and context-specific interactions with stromal and immune cells [21,26]. At the molecular level, TANs frequently exhibit increased expression of molecules associated with tissue retention and activation, including oxidized low-density lipoprotein receptor 1 (OLR1), vascular endothelial growth factor A (VEGFA), CD83, intercellular adhesion molecule 1 (ICAM1), and chemokine receptor C-X-C motif chemokine receptor 4 (CXCR4) [27,28]. In parallel, they show reduced expression of canonical chemokine receptors such as C-X-C motif chemokine receptor 1 (CXCR1) and C-X-C motif chemokine receptor 2 (CXCR2), along with diminished levels of maturation and trafficking markers including prostaglandin-endoperoxide synthase 2 (PTGS2), CD62L, colony-stimulating factor 3 receptor (CSF3R), and Fc gamma receptor IIIb (FCGR3B) [27]. Single-cell transcriptomic studies across diverse malignancies have revealed that TANs do not constitute a uniform population but rather a spectrum of neutrophil states shaped by the immune context of the tumor [29]. These transcriptional programs may emphasize inflammatory activity, promote angiogenesis, or support antigen presentation, and their prevalence has been linked to clinical outcomes [30]. This phenotypic heterogeneity is sustained by neutrophil plasticity: although post-mitotic, neutrophils can be functionally remodeled by tumor-derived cytokines, metabolic stress, and hypoxia [25,31]. Furthermore, TANs can exhibit dynamic trafficking behavior, including reverse migration into the circulation and homing to distant organs, thereby connecting local tumor inflammation to systemic immune remodeling and the conditioning of metastatic niches [22].

### 3.2. Neutrophils in the Tumor Microenvironment: Dual Roles in Disease Promotion and Immune Surveillance

Within the tumor microenvironment, TANs contribute to cancer progression by sustaining inflammation, altering tissue structure, and promoting metastatic dissemination [22]. A central aspect of their protumor activity lies in the secretion of soluble factors and proteolytic enzymes that either directly support malignant cell survival and motility or indirectly influence the behavior of stromal elements [32]. Among these, cytokines and chemokines such as transforming growth factor beta (TGF-β), interleukin 17A (IL-17A), C-C motif chemokine ligand 2 (CCL2), and interleukin 8/C-X-C motif chemokine ligand 8 (IL-8/CXCL8) have been shown to enhance proliferation, invasiveness, and epithelial to mesenchymal transition-like programs, in part through activation of the Janus kinase 2/signal transducer and activator of transcription 3 (JAK2/STAT3) pathway [32,33]. In parallel, TANs support angiogenesis by releasing provascular mediators including prokineticin-2 (Bv8), matrix metalloproteinase 9 (MMP-9), and vascular endothelial growth factor (VEGF), facilitating endothelial cell expansion, sprouting of new vessels, and increased vascular permeability [34,35]. In advanced disease, TANs can further promote metastasis through the formation of NETs, web-like DNA and protein structures enriched with enzymes such as elastase, myeloperoxidase (MPO), and MMP-9 [36]. These NETs can compromise endothelial barriers to aid tumor cell escape into circulation, physically trap circulating tumor cells (CTCs), and shield them from immune-mediated clearance [37,38]. At the same time, tumors may intensify TAN recruitment and NET formation by upregulating chemokine production or rewiring metabolic pathways that increase CXCL ligand expression, reinforcing a feedback loop that facilitates invasion and metastatic seeding [39].

Despite these protumor roles, TANs are not inherently immunosuppressive and can contribute to antitumor immunity under specific conditions that favor cytotoxic activation and cooperation with adaptive immune components [32]. When properly activated, neutrophils are capable of directly damaging tumor cells via oxidative bursts and the release of cytotoxic molecules during degranulation, especially in the presence of antibody- or complement-mediated targeting and when inhibitory checkpoints are not dominant [21,26]. Furthermore, TANs can act as amplifiers of immune responses by producing inflammatory mediators that recruit and activate dendritic cells, cytotoxic T lymphocytes, and other innate immune populations, thereby enhancing tumor immune surveillance. In certain inflammatory contexts, they may even acquire features of antigen-presenting cells, contributing to T-cell priming or functional restoration [21,40]. However, this antitumor potential is highly dependent on the surrounding microenvironment. The balance between activating signals, including type I and type II interferons and opsonizing antibodies, and immunosuppressive factors such as hypoxia, adenosine accumulation, prostaglandin production, dominant TGF-β signaling, and the expression of immune checkpoint ligands, ultimately determines whether TANs support immune-mediated tumor control or become tolerogenic [24,32]. Within this dynamic network, TANs should be understood as flexible innate effectors whose functional trajectory is shaped by the interplay of tumor-derived stressors, stromal context, and ongoing immune interactions throughout the course of disease and treatment [29].

Furthermore, the neutrophil-to-lymphocyte ratio (NLR) has emerged as a simple, inexpensive, and widely accessible biomarker of systemic inflammation with consistent prognostic relevance across multiple solid malignancies. In broad evidence syntheses, elevated NLR has generally been associated with worse overall survival, progression-free survival, and more aggressive disease behavior, supporting the concept that a neutrophil-skewed host response reflects a tumor-promoting inflammatory state coupled with relative impairment of lymphocyte-mediated antitumor immunity [41]. In pancreatic cancer specifically, meta-analytic and clinical studies have shown that higher NLR is associated with poorer outcomes, including after resection and systemic therapy, further linking increased neutrophil abundance to unfavorable tumor biology and immune suppression [42]. Similar observations have also been reported in breast cancer, where elevated pretreatment NLR has been proposed as a marker of inferior prognosis and a potentially useful tool for risk stratification [43]. Collectively, these findings strengthen the biological rationale that neutrophil expansion and neutrophil-dominated inflammatory programs are not merely epiphenomena, but are meaningfully linked to cancer progression and clinical outcome.

### 3.3. Neutrophil-Driven Resistance to Cytotoxic and Immune-Based Cancer Therapies

Clinical and translational studies increasingly associate high densities of intratumoral neutrophils with poor responses to cytotoxic chemotherapy across multiple cancer types, supporting the concept that a neutrophil-rich TME can attenuate drug efficacy [44]. A recurring upstream mechanism involves tumor-derived chemokine signaling that sustains neutrophil recruitment, with C X C motif chemokine ligand 1 (CXCL1) frequently identified as a representative attractant linked to treatment failure in metastatic disease [45]. When cytotoxic agents impose genotoxic and proteotoxic stress on malignant cells, the surrounding immune and stromal context often determines whether this stress results in irreversible cell death or is diverted into a state of tolerance [12]. Neutrophils contribute to this adaptive buffering by modulating the local inflammatory milieu, influencing vascular function and permeability, and reinforcing cytokine networks that favor survival and phenotypic adaptation rather than effective tumor clearance [46].

Beyond conventional chemotherapy, neutrophils can also compromise the efficacy of immune checkpoint inhibitors by sustaining an immunosuppressive tumor ecosystem that weakens cytotoxic T lymphocyte activity [47]. A central mechanism involves neutrophil-driven attrition and functional exhaustion of T cells within tumors, which limits responsiveness to programmed cell death protein 1 and programmed death ligand 1 (PD 1 and PD L1) blockade [48]. Tumor-intrinsic signaling programs can actively recruit and condition neutrophils through pathways converging on VEGF and CXCL networks, resulting in reduced frequencies of activated cluster of differentiation 8 positive (CD8^+^) T cells and enrichment of dysfunctional or depleted T cell states [48,49]. In addition, subsets of tumor-infiltrating neutrophils may express PD L1 and directly suppress T cell activity through checkpoint-dependent interactions [50,51]. This phenotype can be reinforced by tumor-derived growth factors such as granulocyte macrophage colony-stimulating factor (GM CSF) acting through JAK/STAT3 signaling [46]. Complementing checkpoint-mediated suppression, neutrophils can induce apoptosis of CD8^+^ T cells via contact-dependent mechanisms involving mediators such as tumor necrosis factor alpha (TNF alpha) and nitric oxide, further reducing the effector pool required for sustained immunotherapy benefit [52].

A similar framework applies to adoptive cellular therapies, where the major limitation in solid tumors is often not antigen recognition but the hostile microenvironment that constrains T cell persistence and function [53,54]. Neutrophils represent one element of this barrier by participating in a broader suppressive network that includes myeloid-derived suppressor cells (MDSCs), tumor-associated macrophages, stromal components, and regulatory T cells (Tregs) [55]. Together, these elements create a niche that restricts immune cell trafficking, promotes exhaustion-associated transcriptional programs, and dampens cytotoxic effector functions [56]. The cumulative consequence is a reduced capacity of engineered or transferred T cells to expand and maintain antitumor activity [56]. Taken together, these observations position neutrophils as context-dependent modulators of therapeutic response, capable of transforming otherwise effective treatment-induced stress into clinically relevant resistance through coordinated microenvironmental remodeling and suppression of adaptive immunity.

## 4. Neutrophil Extracellular Traps Across Biology and Disease: From Host Defense to Cancer and Therapy Resistance

Neutrophils are fast-acting innate immune cells that serve as the body’s first line of defense against microbial threats by engaging in three interrelated effector strategies. The first involves phagocytosis, a process in which neutrophils engulf bacteria or other pathogens into intracellular vesicles known as phagosomes [57]. These compartments subsequently mature and fuse with granules, a vesicle packed with proteolytic enzymes, creating microbicidal environments enriched with ROS and antimicrobial enzymes that eliminate internalized pathogens [57]. The second mechanism centers on degranulation, whereby neutrophils release cytotoxic molecules such as proteases and oxidants into the extracellular space in a tightly regulated fashion [58]. This release not only aids in pathogen destruction but also modulates the local inflammatory response [59]. The third effector strategy is the formation of NETs, which are lattices of DNA and antimicrobial proteins expelled into the extracellular space to immobilize and neutralize invading organisms [36,58]. While these mechanisms are indispensable for managing acute infections and facilitating wound repair, their prolonged or dysregulated activation can drive tissue damage, sustain chronic inflammation, and contribute to tumor progression by altering immune cell interactions and reshaping the tumor microenvironment [60].

Originally identified in 2004, NETs represent a unique antimicrobial strategy in which neutrophils release decondensed chromatin structures studded with bioactive proteins derived from cytoplasmic granules and the cytosol [61]. In the classical NET formation pathway, often termed NETosis, chromatin decondensation is initiated by enzymatic modification of histones, particularly through the action of PAD4, which catalyzes histone citrullination and facilitates nuclear envelope breakdown [62]. Concurrently, granular enzymes including MPO, neutrophil elastase (NE), proteinase 3 (PRTN3), cathepsin G (CTSG), and MMP-9 translocate into the nucleus and become embedded within the expanding chromatin web [63]. Once released into the extracellular space, this DNA-based scaffold serves not only as a physical barrier to pathogens but also as a concentrated source of cytotoxic agents [36]. However, the same molecular features that confer antimicrobial efficacy also underlie the pathological potential of NETs [36]. When formed in excess or inadequately cleared, NETs can damage host tissues, perpetuate sterile inflammation, trigger autoimmunity, and promote thrombotic events, all of which have been implicated in the pathogenesis of various noninfectious diseases, including cancer and cardiovascular disorders [64].

NETosis is a specialized form of neutrophil activation that culminates in the release of NETs, and it is mechanistically distinct from classical cell death pathways such as apoptosis and necrosis [36]. This process can be initiated by a variety of stimuli relevant to both infectious and sterile inflammatory settings, including microbial components, immune complexes, activated platelets, and proinflammatory cytokines [61]. At the cellular level, NETosis involves a series of coordinated steps beginning with chromatin decondensation and disintegration of the nuclear envelope [62]. During this sequence, granular enzymes such as NE and MPO translocate into the nucleus, where they promote chromatin relaxation and prepare it for extracellular release [63]. Once this chromatin is sufficiently expanded and decorated with antimicrobial proteins, it is expelled from the cell as a web-like DNA scaffold [61]. In lytic forms of NETosis, this is accompanied by permeabilization of the plasma membrane and eventual cell death [36,65]. In the context of cancer, these same pathways are increasingly recognized as being exploited by tumors, particularly within inflamed microenvironments, where NETosis contributes to tissue remodeling, immune evasion, and progression of malignancy [66].

From a mechanistic perspective, NETosis encompasses at least three overlapping pathways, each with distinct kinetics and cellular consequences [61]. The first, often referred to as suicidal or lytic NETosis, is a relatively slow process that requires the generation of ROS via nicotinamide adenine dinucleotide phosphate (NADPH) oxidase, typically in response to strong stimulants like phorbol esters. This pathway culminates in cell lysis after several hours [65,67]. In contrast, vital NETosis permits the release of NETs while preserving neutrophils plasma membrane and ongoing functionality. This form is marked by PAD4-mediated citrullination of histones, vesicular chromatin packaging, and extrusion of nuclear content without immediate cell death [64,68]. A third, less conventional pathway involves mitochondrial NETosis, in which mitochondrial rather than nuclear DNA is released. This can be triggered by mitochondrial ROS, calcium flux, complement activation, or specific autoantibody interactions and tends to occur rapidly, often with minimal disruption of the plasma membrane [36]. Across all forms of NETosis, several molecular markers are used to track its activity and biological relevance. Citrullinated histone H3 (CitH3) serves as a surrogate of PAD4 activity and is closely linked to chromatin remodeling [36]. NE is critical for histone degradation and chromatin relaxation, and its absence severely limits NET formation [69]. MPO, in addition to facilitating chromatin changes, contributes to the proinflammatory properties of NETs [70]. These components together provide mechanistic insight into how a normally protective immune response may, when dysregulated or co-opted by tumors, contribute to chronic inflammation and cancer-associated pathology.

### 4.1. The Dual Biology of NETs: Host Defense Versus Sterile Immunopathology

Neutrophil extracellular traps serve a critical role in innate immunity by enabling neutrophils to immobilize pathogens, concentrate antimicrobial proteins, and initiate communication with other immune cells at sites of infection or injury [61,71]. Through the formation of these chromatin-based scaffolds enriched with enzymes and histones, NETs help coordinate local inflammation and containment of microbial threats [68]. However, when NET formation becomes excessive, persistent, or poorly cleared—particularly in the absence of infection—their function can shift from protective to pathogenic [72]. In sterile inflammatory diseases, extracellular DNA and histones released during NETosis may act as DAMPs, triggering immune activation and serving as autoantigens that perpetuate autoantibody production and chronic inflammation [73]. This mechanism has been implicated in the pathogenesis of autoimmune diseases such as systemic lupus erythematosus (SLE) and rheumatoid arthritis (RA), where NETs contribute to the persistence of self-reactive immune responses [36,73].

Prolonged NET-driven inflammation can also promote tissue remodeling and progressive injury, playing a role in chronic conditions such as atherosclerosis and chronic obstructive pulmonary disease (COPD) [74,75]. In these diseases, NET components can activate nucleic acid-sensing pathways and amplify NF-κB-mediated inflammatory cascades, leading to sustained immune cell recruitment and tissue damage [76]. Importantly, NETs are not merely passive structures; they contain a complex array of bioactive proteins that can influence coagulation, vascular permeability, and immune cell function [61,72]. This biochemical diversity helps explain why NETs have been increasingly implicated in thromboinflammatory disorders and severe viral infections, including coronavirus disease 2019 (COVID-19), where their unchecked formation is associated with endothelial injury, microvascular thrombosis, and heightened inflammatory responses [61]. Taken together, these findings highlight the dual nature of NETs as both defenders and potential drivers of chronic disease, depending on the context, duration, and regulation of their formation.

Neutrophil extracellular traps have gained increasing recognition as modulators of cancer biology, particularly due to their capacity to alter the immune landscape and restructure the tumor–host interface [36]. Within the TME, NETs can create physical and biochemical barriers that interfere with effective immune surveillance [36]. By limiting access of cytotoxic lymphocytes to tumor cells and incorporating checkpoint-active ligands and immunosuppressive mediators into the extracellular matrix, NETs help maintain an environment conducive to immune escape [77]. This mechanistic understanding has prompted preclinical efforts to target NETs therapeutically, including the use of DNase I, which degrades the extracellular DNA scaffold of NETs and thereby facilitates their dismantling and clearance, and these strategies aimed at reducing NET burden or disrupting their structural integrity showing improved responses to immune checkpoint blockade [78]. Inhibiting NETs has been shown to restore T-cell infiltration, reinstate effector functions, and dampen local immunosuppressive signaling [78]. Concurrently, markers of NET activity, such as CitH3, are under investigation as potential biomarkers of inflammation-driven tumor phenotypes, particularly in tumor-adjacent fluids or systemic circulation [79].

At the molecular level, NETs enhance invasion and metastatic spread through their structural DNA scaffold and associated proteolytic cargo [66]. Enzymes embedded within NETs, including NE and MMP-9, can degrade components of the extracellular matrix, exposing hidden ligands that facilitate tumor cell migration and entry into surrounding tissues [80,81,82]. Additional proteases, such as CTSG, have also been implicated in supporting invasive behavior in specific malignancies [82,83]. Moreover, NETs influence endothelial dynamics by increasing vascular permeability, promoting endothelial cell activation, and capturing CTCs within microvessels [61,84]. This process facilitates extravasation and metastatic colonization of distant organs [61]. Tumor cells themselves can detect NET-associated signals through receptors such as Toll-like receptors (TLRs) and the coiled-coil domain containing protein 25 (CCDC25), which drive enhanced motility, survival, and directed migration toward NET-rich regions [36,85]. This bidirectional relationship fosters a positive feedback loop in which tumor-derived cues activate neutrophils, while NETs, in return, augment metastatic fitness.

However, the effects of NETs on tumor progression are not universally protumorigenic and appear to depend heavily on context. In some experimental models, NET formation during chemotherapy has been associated with direct tumor cell cytotoxicity, likely mediated by intense oxidative stress [61,86,87]. Clinical studies have also reported correlations between high NET-associated signatures in tumor effusions and improved outcomes in select patient subgroups. These findings suggest a functional dichotomy, in which the intensity and localization of oxidative and enzymatic activity may steer NET biology toward either tumor suppression or tumor support [88]. For instance, a high burden of ROS may induce tumor cell death, while lower, localized oxidative signaling may promote adaptation, immune evasion, and stromal remodeling [89]. Factors such as tumor histology, local oxygen tension, the oxidative balance between neutrophil-derived ROS and tumor antioxidant defenses, and the enrichment of PD-L1 or other immune-modulatory components within NETs all contribute to determining whether NET activity ultimately constrains or facilitates malignancy [90,91].

### 4.2. NETosis-Driven Chemoresistance: Clinical Associations and Translational Implications

An expanding body of clinical evidence indicates that NET formation, or NETosis, may serve as a marker of treatment resistance in various malignancies [92,93,94]. Studies in locally advanced rectal cancer and metastatic renal cell carcinoma have linked NET-related molecular patterns to diminished chemotherapy response [92,93]. Similar associations have been observed in lung adenocarcinoma, where NET-associated transcriptomic and proteomic signatures correlate with poor survival and therapeutic failure [94,95]. In colorectal cancer, elevated levels of circulating CitH3, a marker of NETosis, have been found in patients with unfavorable outcomes, supporting its potential use for risk stratification and longitudinal monitoring [96]. Evidence from pediatric osteosarcoma further underscores the relevance of NET burden, with high levels associated with poor responses to neoadjuvant treatment and reduced survival [97]. These findings suggest that NETs may reflect an underlying inflammatory tumor state that is inherently less susceptible to cytotoxic therapy.

From a mechanistic standpoint, NETosis appears to contribute to chemoresistance not through a single linear pathway, but by broadly reprogramming the TME [98,99]. NET-derived enzymes and structural components can interact with stromal and vascular cells, creating a niche that buffers against therapeutic stress and supports tumor cell survival [100,101]. In pancreatic ductal adenocarcinoma (PDAC), NETs have been shown to activate pancreatic stellate cells, which are known to mediate desmoplasia and drug resistance, thereby reinforcing stromal barriers to therapy [102,103]. In addition, interactions between tumor cells and activated platelets may trigger NET formation in circulation, linking coagulation pathways with immune evasion and metastatic dissemination [66,104,105]. NETs can also disrupt endothelial and lymphatic integrity, increasing vascular permeability and potentially altering drug distribution [106,107]. At the same time, NET-enriched environments may promote recruitment of regulatory immune cells and suppress effector lymphocyte function, thus contributing to a broader immunosuppressive milieu that limits the efficacy of both chemotherapy and immunotherapy [108].

An additional dimension is the possibility that chemotherapy itself can initiate NETosis, establishing a self-reinforcing cycle of inflammation and resistance [98,109]. Dying tumor cells release damage-associated signals such as adenosine triphosphate (ATP), which can activate inflammasome pathways in adjacent cells and promote production of interleukin 1 beta (IL-1β), a cytokine known to drive neutrophil activation and NET release [109,110]. These NET-rich microenvironments, in turn, can suppress the cytotoxic activity of T cells, further impairing immune-mediated tumor clearance [111,112]. Experimental models support this sequence of events, showing that inhibition of NETosis—either through degradation of extracellular chromatin or by targeting regulatory enzymes such as PAD4—can restore chemotherapy and immune checkpoint efficacy [78,113]. Other NET-associated suppressive mechanisms include enhanced arginase 1 activity that inhibits T-cell proliferation, and IL-17-mediated neutrophil recruitment that perpetuates NETosis while excluding cytotoxic lymphocytes [114,115]. Together, these findings support a model in which therapy-induced inflammation promotes NET formation, and NETosis, in turn, remodels the TME to favor tumor cell plasticity, immune evasion, and durable resistance to treatment.

## 5. ChemoNETosis: A Treatment-Evoked NET Program That Limits Chemotherapy Efficacy

ChemoNETosis refers to a form of treatment-induced inflammation in which chemotherapy stimulates NET formation, ultimately contributing to reduced therapeutic efficacy [99,109]. Rather than acting exclusively on tumor cells, chemotherapy also reshapes the cytokine and chemokine landscape within the TME, generating gradients that recruit and activate neutrophils in susceptible regions, including metastatic niches [116,117]. Key mediators in this process include CXCL1 and CXCL5, which signal through CXCR2, along with proinflammatory cytokines such as IL-1β that promote NETosis [116,117]. Once released, NETs contribute to tumor progression by inducing phenotypic plasticity and drug tolerance [118]. At the mechanistic level, this includes the activation of TGF-β signaling, which facilitates epithelial to mesenchymal transition (EMT), as well as broader transcriptional reprogramming that reinforces EMT-associated gene expression profiles [118,119]. Importantly, preclinical studies have shown that blocking ChemoNETosis, either by targeting PAD4 to prevent chromatin decondensation or by administering DNase to degrade extracellular chromatin, can restore sensitivity to chemotherapy, suggesting a direct and modifiable link between NET formation and treatment resistance [116]. Emerging evidence suggests that NETs may facilitate metastasis not only by remodeling TME locally, but also by trapping circulating tumor cells (CTCs) in the bloodstream and promoting their adhesion at distant organ sites. Experimental studies have shown that CTCs can become physically sequestered within NET structures under both static and flow conditions, thereby enhancing early metastatic arrest, while NET disruption with DNase I or NE inhibition reduces tumor-cell adhesion and metastatic burden [120]. Mechanistically, NET-derived DNA can be sensed by the tumor-cell surface receptor CCDC25, which activates integrin-linked kinase (ILK)–β-parvin-dependent cytoskeletal remodeling to promote migration, adhesion, and colonization [121]. Accordingly, NETs are increasingly viewed as adhesive and chemotactic scaffolds that support CTC survival, extravasation, and metastatic outgrowth [61]. Although direct proof that chemotherapy-induced NET formation (chemoNETosis) exploits this pathway remains limited, the concept is strongly supported by recent findings showing that anticancer therapy can increase NET-DNA accumulation in breast cancer and metastatic organs, while NET–CCDC25 signaling contributes to treatment resistance and metastatic progression. Together, these observations support the possibility that chemoNETosis may also enhance metastatic dissemination by facilitating CTC trapping and adhesion at secondary sites.

Evidence for this mechanism has been most clearly demonstrated in breast cancer, pancreatic ductal adenocarcinoma, and colorectal cancer, where ChemoNETosis is identified as a relevant factor influencing therapeutic outcomes. The following sections will therefore examine these malignancies in greater detail, highlighting the available evidence that supports a critical role for ChemoNETosis within their tumor microenvironment and its impact on treatment response.

## 6. ChemoNETosis in Breast Cancer: Therapy-Evoked Innate Programs That Reshape Response

Breast cancer remains the most frequently diagnosed cancer in women worldwide and a major contributor to cancer-related mortality, with metastatic disease responsible for most deaths [1,122]. While advances in early detection and the integration of surgery, radiotherapy, and systemic treatments have improved survival in patients with localized tumors, clinical management of advanced disease is complicated by significant biological heterogeneity [1,123,124]. This heterogeneity encompasses variations in histological subtypes, hormone receptor expression, proliferation rates, and patterns of metastatic spread [125]. As a result, therapeutic responses vary widely, and breast cancer continues to exert a substantial global health burden, with notable disparities in incidence, access to care, and outcomes across geographic and socioeconomic lines.

In the metastatic setting, systemic cytotoxic chemotherapy remains a cornerstone of treatment aimed at controlling disease progression [126]. However, long-term benefit is frequently undermined by the development of chemoresistance [127]. Emerging evidence suggests that resistance is not solely a consequence of tumor cell-intrinsic alterations, such as genetic mutations or epigenetic changes, but is also shaped by the broader TME [128,129]. Host-derived elements, including growth factors, cytokines, and extracellular matrix components, can mitigate the impact of chemotherapeutic stress, activate survival signaling, and support adaptive phenotypic changes that allow residual malignant cells to endure and eventually proliferate [130,131]. Among these components, inflammatory myeloid cells—particularly monocytes and macrophages—play a central role by producing prosurvival mediators and remodeling tissue structure in response to therapy [132]. Moreover, chemotherapy itself may potentiate these effects by inducing chronic inflammation, thereby reinforcing protective features of the TME and accelerating the progression from initial therapeutic response to overt resistance and disease relapse [133].

### 6.1. Neutrophils and NETosis in Breast Cancer: Context-Dependent Drivers of Progression

Breast cancer progression is now increasingly viewed as a dynamic ecosystem-level process in which the TME plays a central role by promoting malignant growth, sustaining intratumoral heterogeneity, and limiting therapeutic efficacy [134]. Within this complex environment, neutrophils have emerged as influential but previously underrecognized contributors to disease evolution [135]. Their functional impact is highly context dependent, ranging from direct cytotoxicity against tumor cells to facilitation of cancer progression through the release of proinflammatory signals, proteolytic enzymes, and angiogenic mediators, as well as the suppression of adaptive immune responses [136,137]. These effects are shaped by local concentrations of cytokines, chemokines, and metabolic stressors, which collectively determine neutrophil polarization and effector function [136]. Through their ability to modulate tissue architecture, promote epithelial to mesenchymal transition, and influence the survival and behavior of therapy-tolerant clones, neutrophils integrate innate inflammatory responses into the molecular framework that drives disease persistence and resistance in breast cancer [138].

NETosis is increasingly recognized as a relevant inflammatory pathway in breast cancer, with evidence suggesting that the route of NET formation—whether suicidal, vital, or mitochondrial—may shape distinct aspects of tissue remodeling and immune modulation [139]. Observational studies have reported that NET activity is more pronounced in triple-negative breast cancer (TNBC) compared to estrogen receptor-positive (ER-positive) subtypes, and elevated NET burden has been associated with larger primary tumors, higher proliferation rates, and greater lymph node involvement [139,140]. Functionally, NETs appear to remodel the breast TME by reprogramming macrophages toward immunosuppressive phenotypes, pro-M2 phenotype, and enhancing the invasive behavior of cancer-associated fibroblasts [139]. These effects establish conditions that support local tumor progression and reflect a broader conceptual shift in which NETosis is no longer viewed as a passive consequence of inflammation, but rather as an active driver of cellular communication and tumor aggressiveness, particularly in biologically aggressive breast cancer subtypes.

In parallel, components of NETs are being evaluated as candidate biomarkers for disease activity and metastatic potential in breast cancer [139]. Circulating markers such as CitH3, NE bound to DNA, and cfDNA are under investigation as minimally invasive indicators of NETosis intensity [139]. From a therapeutic perspective, experimental models have shown that targeting NET formation or disrupting the extracellular DNA scaffold can limit metastatic progression [141,142]. Two promising strategies include deoxyribonuclease I (DNase I), which enzymatically degrades extracellular chromatin to destabilize NETs, and inhibition of PAD4, an enzyme required for histone citrullination and chromatin decondensation [141,143]. While these approaches are still in early stages of development, they represent a focused method to interfere with defined inflammatory effectors rather than broadly suppressing innate immunity, potentially preserving essential host defense functions while mitigating pro-tumor inflammation.

On a molecular level, NETs influence multiple hallmarks of breast cancer progression, including epithelial plasticity, metastatic spread, neovascularization, and resistance to therapy [142]. Exposure to NET-rich environments has been shown to induce a shift in tumor cells toward a mesenchymal-like state, marked by downregulation of epithelial adhesion molecules and upregulation of motility-associated genes, thereby enhancing invasive potential [139]. Within the circulation and at distant endothelial sites, NETs function as adhesive matrices that trap CTCs and facilitate their extravasation through integrin-mediated adhesion and activation of cytoskeletal regulators such as coiled-coil domain-containing protein 25 (CCDC25) and its downstream targets, including integrin-linked kinase (ILK), beta-parvin, and small GTPases RAC1 and CDC42 [87,139,144]. Proteolytic enzymes embedded in NETs, such as NE and MMP-9, degrade ECM components to ease tissue penetration, while NET-associated factors, including VEGF, promote angiogenesis stimulate endothelial remodeling and vascular permeability [36,87]. Concurrently, NETs can shape the immune landscape by favoring immunosuppressive myeloid responses, impairing dendritic cell maturation, and upregulating immune checkpoints such as PD-L1, leading to diminished cytotoxic T-cell activity [66,145]. Finally, cytotoxic chemotherapy may inadvertently amplify NET formation through IL-1β-driven inflammation, establishing a microenvironment that activates TGF-β-linked epithelial to EMT and enhances the survival of therapy-resistant tumor cell populations [146].

### 6.2. ChemoNETosis as a Facilitator of Therapy Resistance in Breast Cancer Metastasis

ChemoNETosis in breast cancer refers to a treatment-induced inflammatory cascade in which cytotoxic chemotherapy alters the metastatic microenvironment, leading to the recruitment and activation of neutrophils and ultimately triggering the formation of NETs [99,116]. Rather than being a direct response of neutrophils to chemotherapy, this process unfolds through changes in cytokine and chemokine signaling within the tumor tissue, particularly in metastatic niches such as the lungs [116]. These signaling alterations create a permissive inflammatory context that supports NETosis, positioning NET formation as a biologically active process that contributes to resistance, rather than a passive marker of inflammation [98,116].

In murine models of breast cancer with pulmonary metastases, platinum-based agents and anthracycline–cyclophosphamide combinations have been shown to increase neutrophil accumulation in metastatic lung lesions while simultaneously reducing the efficacy of chemotherapy [85,116]. This recruitment is driven largely by the upregulation of CXCL1 and CXCL5, which act through the CXCR2 on neutrophils [116]. Notably, the trigger for NET formation originates not from direct exposure of neutrophils to cytotoxic drugs, but from tumor cell crosstalk under stress conditions, where damaged and surviving cancer cells coordinate a local immune response that initiates NETosis [116,147].

A key mechanistic pathway involves the release of ATP from dying tumor cells, which serves as a DAMP. ATP is sensed by adjacent surviving cancer cells via purinergic P2X7 receptors, leading to activation of the NOD-, LRR- and pyrin domain-containing protein 3 (NLRP3) inflammasome [116]. This, in turn, activates caspase-1, which processes pro-interleukin 1 beta (pro–IL-1β) into its mature, bioactive form [116,148,149]. Tumor-derived IL-1β acts as a strong inducer of NETosis in recruited neutrophils, establishing a feedback loop in which chemotherapy generates inflammatory conditions that promote neutrophil activation and extracellular trap release within metastatic sites [66,116].

Once released, NETs function as extracellular platforms that concentrate cytokines and proteases in close proximity to cancer cells [66,142]. Two components are especially relevant for the resistance phenotype in breast cancer metastases: the integrin αvβ1, which binds latent TGF-β within the NET matrix, and MMP-9, which converts this latent TGF-β into its active form [116]. While many tumors produce TGF-β, it is generally secreted in a latent state that requires extracellular processing. By capturing and activating this cytokine, NETs localize TGF-β signaling to the tumor–stromal interface, intensifying its effects on nearby tumor cells and fostering resistance through precise microenvironmental remodeling [116,150].

Activated TGF-β signaling engages its receptor on tumor cells, initiating SMAD2 phosphorylation and downstream transcriptional programs associated with EMT [151,152]. In metastatic lung lesions, this translates into the loss of epithelial markers such as claudin-1 and upregulation of mesenchymal traits, including increased expression of N-cadherin [116]. These changes are consistent with a more motile and therapy-tolerant phenotype. EMT not only facilitates invasion and dissemination but also supports a broader range of cellular states that can survive chemotherapy, highlighting how NET-driven signaling contributes directly to the development of chemoresistant tumor subpopulations [118].

Beyond its role in resistance, chemoNETosis also impacts treatment-related toxicity [116]. In experimental models, cisplatin has been associated with the accumulation of NET-forming neutrophils in the kidneys, contributing to nephrotoxicity [116]. NET-derived histones and enzymes may exacerbate tubular injury by engaging innate immune sensors, and pharmacologic disruption of NETs has been shown to attenuate renal dysfunction [116,153]. These findings suggest that NETosis is not only a mechanism of tumor adaptation but also a contributor to collateral tissue damage, underscoring the therapeutic potential of targeting NETs to simultaneously enhance treatment efficacy and reduce toxicity.

In the neoadjuvant setting, additional mechanisms have been proposed to explain NET-associated vascular injury [100,147]. Patients receiving docetaxel and carboplatin have demonstrated elevated levels of circulating NET markers, along with increased biomarkers of endothelial damage [147]. This injury appears to be linked to metabolic reprogramming of neutrophils under chemotherapy, particularly through upregulation of solute carrier family 11 member 1 (Slc11a1), which increases intracellular ferrous ion levels [147,154]. The elevated iron enhances ROS generation and supports PAD4-mediated chromatin remodeling, facilitating NET formation [155,156]. These NETs contribute to endothelial activation and glycocalyx degradation, as evidenced by elevated levels of von Willebrand factor and syndecan-4, creating a prothrombotic and inflammatory vascular phenotype that can complicate surgical outcomes [84,147].

**Figure 1:**
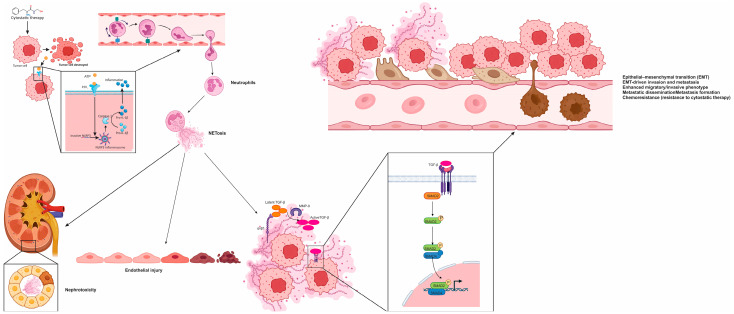
Conceptual overview of chemoNETosis in breast cancer linking chemotherapy-induced danger signaling to NET formation, EMT, chemoresistance, and treatment-associated toxicity. Cytotoxic chemotherapy injures tumor cells and promotes the release of danger signals, including extracellular ATP. ATP is sensed by neighboring surviving tumor cells via the purinergic P2X7 receptor, which supports activation of the NLRP3 inflammasome, caspase-1, and the maturation of IL-1β. In parallel, chemotherapy alters chemokine cues within metastatic sites (e.g., increased CXCL1 and CXCL5), favoring CXCR2-dependent neutrophil recruitment and accumulation, particularly in lung metastatic lesions. Within this inflammatory context, IL-1β acts as a strong trigger for neutrophil activation and NETosis, resulting in NET deposition in close proximity to tumor cells. Once formed, NETs function as a bioactive extracellular scaffold that concentrates signaling mediators and remodels the tumor–stromal interface. A key feature highlighted here is the capture of latent TGF-β within the NET matrix (via integrin αvβ1) and its activation through MMP-9, thereby amplifying local TGF-β signaling. Activated TGF-β engages tumor cell receptors and propagates canonical SMAD signaling, promoting EMT-associated transcriptional programs that support invasion, metastatic dissemination, and reduced sensitivity to cytotoxic therapy. The schematic also summarizes clinically relevant collateral effects attributed to chemoNETosis, including endothelial injury with a pro-inflammatory/prothrombotic vascular phenotype and NET accumulation in the kidney that may contribute to nephrotoxicity.

Taken together, emerging evidence reframes chemotherapy resistance in metastatic breast cancer as an ecosystem-level phenomenon in which cytotoxic stress reshapes the tumor microenvironment, recruits neutrophils, and under permissive cytokine and chemokine cues drives NET formation with direct pro-metastatic and pro-resistance functions. In this setting, chemoNETosis is best understood as a two-step, checkpointed cascade: first, chemotherapy-associated DAMP release, particularly ATP, activates the P2X7–NLRP3–caspase-1 signaling axis in stressed tumor cells, amplifying IL-1β production and thereby promoting NETosis in recruited neutrophils; second, NET scaffolds act as biochemical platforms that spatially concentrate and activate latent TGF-β through αvβ1 integrin and MMP-9, triggering SMAD-dependent EMT programs that favor therapy tolerance and metastatic persistence. Beyond these effects, NETs also remodel stromal and immune networks, trap circulating tumor cells, facilitate extravasation, support angiogenic remodeling, and dampen antitumor immunity, while parallel mechanisms involving Slc11a1, iron metabolism, and ROS may further contribute to NET-associated vascular and endothelial injury, particularly in the neoadjuvant setting. Circulating NET-related components, including CitH3-, NE–DNA-, and cfDNA-based readouts, are therefore being explored as minimally invasive biomarkers of disease activity, whereas therapeutic targeting of NETs may represent a dual opportunity to enhance treatment efficacy while reducing collateral toxicities. These interconnected molecular checkpoints, biological consequences, and therapeutic implications are schematically summarized in Figure 1 and further condensed in Table 1.

## 7. ChemoNETosis in Colorectal Cancer: Mechanistic Insights into How Cytotoxic Stress Modulates Neutrophil Function and Tumor Fate

Colorectal cancer (CRC) continues to represent a significant global health burden, ranking among the most commonly diagnosed malignancies and remaining a leading cause of cancer-related mortality worldwide [1]. This disease typically arises through a well-characterized multistep sequence, beginning with benign adenomatous polyps that gradually accumulate genetic and epigenetic alterations, eventually progressing to invasive carcinoma [157]. Although substantial progress has been made in treatment strategies, the prognosis for many patients remains limited due to the high frequency of metastatic disease [158]. A notable proportion of individuals are diagnosed with synchronous metastases, and survival rates decline markedly once distant spread is established [159,160]. Detecting metastatic potential at an early stage is therefore crucial for optimizing clinical outcomes, yet current diagnostic approaches have key limitations. Conventional cross-sectional imaging techniques often fail to identify micrometastases, while more sensitive methods such as positron emission tomography combined with computed tomography (PET-CT) and circulating tumor DNA (ctDNA) analysis, though promising, may be hindered by high cost and limited accessibility [161,162]. These challenges highlight the urgent need for reliable, affordable biomarkers and integrated risk assessment tools that can enhance early prediction of metastatic progression and support more personalized, timely therapeutic decision-making in CRC.

Systemic chemotherapy remains a fundamental component in the management of metastatic colorectal cancer (mCRC), where treatment aims include reducing tumor burden, delaying further metastatic progression, alleviating symptoms, and extending survival [158]. These regimens are often administered alongside targeted therapies, guided by the molecular characteristics of the tumor [163]. However, the long-term effectiveness of chemotherapy is frequently undermined by either an initial lack of response or the eventual emergence of chemoresistance [164]. This therapeutic limitation reflects the complex interplay between tumor cell-intrinsic heterogeneity and extrinsic factors within the TME [165]. Within a single patient, spatially and temporally distinct subclonal populations may vary in their capacity for drug uptake, DNA repair, apoptotic signaling, and adaptation to cellular stress [166]. Simultaneously, the surrounding stromal and immune cells influence local cytokine levels, metabolic constraints, and vascular architecture, all of which can reduce the impact of cytotoxic agents [167]. Consequently, treatment responses vary widely among patients, both in magnitude and durability, underscoring the need to develop and validate predictive biomarkers that can inform treatment choices, minimize unnecessary toxicity, and support the implementation of individualized therapeutic approaches in mCRC [168].

### 7.1. Neutrophils and NETs in Colorectal Cancer: Inflammation-Conditioned Programs Shaping the Tumor Microenvironment

In colorectal cancer, neutrophils are among the most abundant immune cells within the TME and exhibit context-dependent functions that reflect the broader inflammatory milieu in which CRC arises [167]. The disease encompasses hereditary syndromes, sporadic cases, and colitis-associated cancer, all of which share a common thread of inflammation as a contributing factor to tumorigenesis and progression. Inflammation in CRC may precede malignant transformation, be driven by the tumor itself, or emerge in response to therapeutic intervention [169,170,171]. In each scenario, innate immune activation frequently promotes tumor-supportive pathways while suppressing effective adaptive immunity, fostering an immunosuppressive niche [172]. Within this framework, neutrophils can reinforce pro-tumor inflammation through the release of cytokines, proteolytic enzymes, and reactive oxygen species, although under specific conditions they may also exert antitumor functions [173]. One effector mechanism of growing interest is the formation of NETs. NETs not only remodel tissue architecture but also modulate interactions among immune cells, with potential implications for tumor invasion and immune evasion [174,175].

From a translational perspective, targeting neutrophil biology in CRC presents both promise and complexity [176]. Direct depletion of neutrophils is not feasible due to the risk of immunosuppression and neutropenia, and the lack of clearly defined neutrophil subsets limits precision-based strategies [177]. Consequently, therapeutic interest has shifted toward modulating neutrophil recruitment and function. The C X C chemokine receptor 1 and 2 (CXCR1/2) and IL-8 axis plays a central role in mediating neutrophil chemotaxis and activation in CRC, and is considered a rational target, along with other regulators such as granulocyte colony-stimulating factor (G-CSF), TGF-β, and VEGF [173,178,179]. Experimental data also suggest that neutrophil-derived mediators may interact with JAK/STAT signaling during the transition from colitis to cancer. Moreover, neutrophil-enriched tumors may exhibit altered responses to immunotherapy, potentially due to the expression of immune checkpoint molecules [176]. For instance, neutrophils can inhibit T-cell and natural killer (NK) cell function through cytotoxic T lymphocyte-associated protein 4 (CTLA-4) and PD-1/PD-L1 pathways [180,181]. Tumor-secreted cytokines can further upregulate PD-L1 on neutrophils via STAT3-dependent mechanisms, suggesting that the efficacy of immune checkpoint blockade may be influenced by the composition and activation state of the neutrophil infiltrate [176,182]. Emerging preclinical approaches include inhibition of PAD4 to modulate neutrophil effector responses and enhance radiosensitivity, as well as targeting microbial influences on neutrophil function, where bacterial-induced immune checkpoint expression may be reversed by combining antimicrobial therapy with immunotherapy [183,184].

In colorectal cancer, NETs have been detected in both primary tumors and metastatic lymph nodes, and elevated levels of NET-related biomarkers have been reported in patients, underscoring their presence in human disease beyond experimental models [175,185,186]. Clinically, recurrence and metastasis remain common challenges even after seemingly curative surgery [187]. Emerging evidence suggests that systemic or perioperative inflammation may generate a favorable microenvironment for residual tumor cells to survive and re-emerge [188]. Within this context, NET formation has been proposed as a mechanistic bridge linking transient inflammatory events, such as surgical trauma or infection, to tumor relapse, including at resection margins or distant sites [189].

At the molecular level, various stimuli within CRC tissue can initiate NETosis, often converging through chemokine-driven amplification loops [190,191]. Polyphosphates released from mast cell-rich inflammatory zones have been shown to stimulate NET formation in ex vivo models of colorectal carcinoma [175,192]. Another pathway involves the oncogene KRAS, frequently mutated in CRC. Tumor cells harboring KRAS mutations can modulate their microenvironment through the release of extracellular vesicles, which may transfer mutant KRAS protein to neutrophils, promoting their recruitment and NET release via IL-8, also known as CXCL8 [176,177]. IL-8 engages neutrophils through its receptors, CXCR1 and CXCR2, activating intracellular signaling cascades involving Src, extracellular signal-regulated kinase (ERK), and p38 mitogen-activated protein kinase (p38 MAPK), all of which contribute to NETosis [71,175]. NET-rich environments can, in turn, activate nucleic acid-sensing pathways in surrounding stromal and immune cells, establishing a feed-forward inflammatory circuit [61]. Additionally, IL-8-responsive myeloid-derived suppressor cells (MDSCs) that express CXCR1/2 may also participate in the formation of extracellular DNA scaffolds, further broadening the range of cellular contributors to NET-like structures in CRC [193].

Once established, NETs can promote both local tumor growth and distant metastasis through mechanisms that are not directly cytotoxic but instead facilitate tumor dissemination [194,195]. These structures can trap CTCs within microvascular beds of target organs such as the liver and lungs, enhancing their adhesion to endothelial surfaces and improving the efficiency of extravasation and colonization [175,194]. In the hepatic metastatic niche, NETs have been observed to entrap CTCs without inducing cell death, while simultaneously enriching the local milieu with inflammatory mediators like IL-8, which in turn recruits additional neutrophils and perpetuates NET formation [175,177]. Adhesion molecules such as carcinoembryonic antigen-related cell adhesion molecule 1 (CEACAM1) present within NETs may further support tumor–NET interactions and direct homing of cancer cells to secondary sites [196]. Surgical stress appears to intensify these effects: animal studies and clinical observations have shown that operative trauma enhances peritoneal adhesion and the growth of CRC cells via CXCR2-dependent pathways. Furthermore, increased NET formation following liver resection for metastatic CRC has been correlated with worse postoperative outcomes. Importantly, experimental interventions that degrade extracellular DNA, such as DNase administration, have been shown to reduce metastatic burden and prevent postoperative disease progression in preclinical CRC models, highlighting NETosis as a therapeutically actionable target at the interface of inflammation, surgical stress, and metastasis [197].

### 7.2. ChemoNETosis in Colorectal Cancer: When Innate Effector Programs Reinforce Cytotoxic Therapy

A mechanistically distinct variant of chemotherapy-induced neutrophil extracellular trap formation, or ChemoNETosis, has been identified in murine models of CRC, in which NETosis exerts an antitumor rather than a resistance-promoting effect [198]. In this specific context, the combination of the glutaminase inhibitor CB-839 with the cytotoxic agent 5-fluorouracil (5-FU) was found to selectively enhance interleukin 8 (IL-8) production in CRC harboring phosphatidylinositol 4,5-bisphosphate 3-kinase catalytic subunit alpha (PIK3CA) mutations [198]. The resulting chemokine gradient recruits neutrophils into the tumor microenvironment. This finding challenges the conventional view of NETosis as uniformly detrimental and instead highlights its functional plasticity [198]. The biological outcome of NET formation appears to depend on the upstream signals that elicit it, the molecular composition of the NET scaffold, and the way tumor cells respond to those extracellular cues.

On a molecular level, this drug combination initiates a sequence linking oxidative stress to chemokine signaling [198]. The treatment enhances ROS within tumor cells, leading to activation of the nuclear factor erythroid 2-related factor 2 (NRF2) transcriptional program [198]. NRF2 then drives increased IL-8 gene expression, further promoting neutrophil chemotaxis into the tumor. Once neutrophils are present, the oxidative stress within the tumor microenvironment promotes ROS accumulation within these immune cells, facilitating NET release [199,200]. This NETosis process is dependent on chromatin remodeling mediated by PAD4, as pharmacologic inhibition of PAD4, for example using GSK484, was shown to suppress NET formation under this therapeutic regimen [198]. Collectively, these findings delineate a coherent pathway: chemotherapy induces redox stress, which activates NRF2, NRF2 enhances IL-8 production, IL-8 recruits neutrophils, and PAD4-dependent NETosis ensues within the tumor bed.

**Figure 2:**
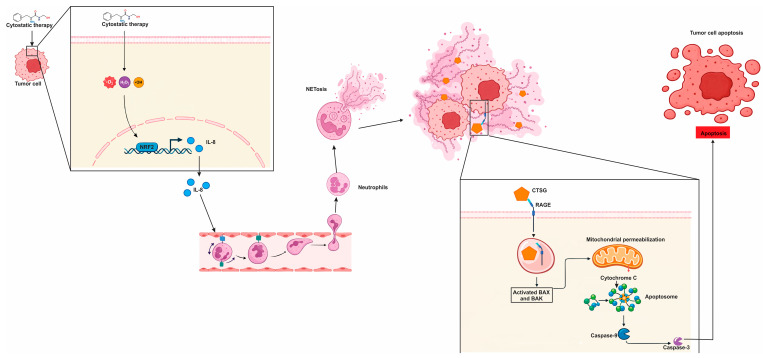
Antitumor chemoNETosis in colorectal cancer: CB-839/5-FU drives an NRF2–IL-8 axis that recruits neutrophils and promotes PAD4-dependent NETosis, enabling NET-borne CTSG to trigger tumor-cell apoptosis. The schematic illustrates a mechanistically distinct, treatment-induced NET program described in murine CRC models, in which NETosis contributes to tumor control rather than chemoresistance. Cytostatic therapy combined with glutaminase inhibition increases oxidative stress in tumor cells, activating NRF2 and enhancing IL-8 expression and secretion. The resulting IL-8 gradient supports neutrophil recruitment into the tumor microenvironment, where oxidative cues promote neutrophil activation and PAD4-mediated chromatin remodeling required for NET release. NETs deposited in the tumor bed carry the serine protease cathepsin G (CTSG), which can bind and enter CRC cells via RAGE. Internalized CTSG cleaves 14-3-3 proteins, relieving inhibitory constraints on pro-apoptotic signaling and facilitating BAX/BAK activation, mitochondrial outer membrane permeabilization, cytochrome c release, apoptosome formation, and downstream caspase activation, culminating in tumor-cell apoptosis. Overall, the figure highlights a context-dependent scenario in which chemotherapy-induced NET formation can act as an effector arm of antitumor immunity, particularly in genetically defined settings such as PIK3CA-mutant CRC.

In this model, the cytotoxic effects of NETs are attributed primarily to CTSG, a serine protease embedded in the NET matrix [198]. Rather than acting solely on extracellular matrix components, CTSG functions as a direct pro-apoptotic effector. It enters CRC cells via the receptor for advanced glycation end products (RAGE), and once internalized, cleaves members of the 14-3-3 protein family. This cleavage event releases inhibitory constraints on apoptotic signaling, allowing Bcl-2-associated X protein (BAX) to translocate to mitochondria and initiate programmed cell death [86,98]. These findings establish a mechanistic link between NET cargo and tumor cell apoptosis. Clinically, this antitumor axis is supported by data showing that higher NET levels in post-treatment tumor samples correlate with improved progression-free survival, particularly in the context of PIK3CA-mutant CRC [198]. These observations suggest that, under defined metabolic and genetic conditions, ChemoNETosis may serve as a therapeutically beneficial effector mechanism rather than an impediment to successful chemotherapy. These relationships are summarized in Figure 2, which depicts this CRC-specific form of chemoNETosis as a redox-to-chemokine cascade that culminates in an antitumor NET effector function.

In summary, colorectal cancer exemplifies how cytotoxic stress and inflammation can reprogram neutrophil behavior in ways that meaningfully shape tumor fate, from metastatic dissemination to therapy response. Within the CRC tumor microenvironment, neutrophils are abundant and highly plastic, with NETosis emerging as a key effector program that can remodel stromal–immune crosstalk, facilitate immune evasion, and provide DNA-based scaffolds that capture circulating tumor cells and promote organ-specific seeding—effects that may be amplified by perioperative inflammatory surges and surgical stress. Translationally, this has redirected therapeutic interest away from neutrophil depletion and toward selective modulation of recruitment and effector function (notably the IL-8/CXCR1/2 axis and PAD4-dependent chromatin remodeling), alongside strategies that disrupt extracellular NET scaffolds (e.g., DNase) to reduce metastatic burden and postoperative progression. Importantly, CRC also offers a mechanistically distinct “beneficial” chemoNETosis paradigm: in PIK3CA-mutant models, CB-839 plus 5-FU induces ROS-driven NRF2 activation in tumor cells, increases IL-8 to recruit neutrophils, and triggers PAD4-dependent NET release; here, NET cargo (CTSG) can enter tumor cells via RAGE, cleave 14-3-3 proteins, enable BAX mitochondrial translocation, and promote apoptosis, with higher post-treatment NET levels correlating with improved progression-free survival. A concise, clinically oriented synthesis of these dual NET programs—pro-metastatic scaffolding versus genotype- and context-restricted antitumor chemoNETosis—is provided in Table 2.

## 8. ChemoNETosis in Pancreatic Ductal Adenocarcinoma: Linking Cytotoxic Injury to Innate Immune Remodeling and Therapeutic Failure

Pancreatic ductal adenocarcinoma, the most prevalent form of pancreatic cancer, continues to rank among the most lethal malignancies, largely due to its asymptomatic early course, aggressive biological behavior, and resistance to current treatment modalities [201]. Early-stage disease often goes undetected, as there are no widely implemented screening tools for individuals at average risk, and clinical symptoms typically emerge only once the tumor has progressed to a locally advanced or metastatic stage [202]. As a result, most patients are diagnosed too late for surgical resection with curative intent, and even when multimodal treatment—including surgery, systemic chemotherapy, radiation, and targeted therapies—is employed, the overall prognosis remains dismal [203]. Systemic chemotherapy remains the cornerstone of treatment in both operable and inoperable cases, yet five-year survival rates for PDAC remain in the low double digits [203]. These sobering statistics emphasize the critical need for earlier detection strategies and more effective therapeutic approaches tailored to the underlying molecular features of the disease [204].

Systemic chemotherapy remains the central component of treatment for advanced PDAC, yet durable responses are rare due to the rapid development of resistance driven by both tumor-intrinsic factors and the complex TME [205,206,207]. In addition to genetic and epigenetic alterations within cancer cells, resistance is further reinforced by the presence of stem-like tumor cell subsets and a desmoplastic TME characterized by extensive fibrotic stroma, aberrant vasculature, and profound immunosuppression. These features act together to impair drug delivery, limit immune activation, and attenuate cytotoxic effects [205,208,209]. Even in patients who undergo resection with curative intent, recurrence is common, reflecting the systemic nature of PDAC biology and the necessity for adjuvant chemotherapy in most cases [210]. The combination of gemcitabine and nanoparticle albumin-bound paclitaxel (nab-paclitaxel) has demonstrated clinical benefit over gemcitabine monotherapy, including in the postoperative setting; however, overall response rates remain modest and disease progression typically occurs within a few months [211]. These observations underscore that chemoresistance in PDAC is not simply a result of cellular escape mechanisms but arises from a coordinated network of interactions among tumor cells, stromal components, and immune elements that collectively define the pharmacologic landscape and constrain the efficacy of standard regimens such as gemcitabine and nab-paclitaxel [205].

### 8.1. Neutrophils and Extracellular Trap Formation in the Tumor Microenvironment of Pancreatic Ductal Adenocarcinoma

In pancreatic ductal adenocarcinoma, neutrophils consistently emerge as key immune players associated with aggressive disease biology, with their enrichment in the TME correlating with poorer clinical outcomes and reduced responsiveness to therapies [212,213]. Rather than serving as passive immune infiltrates, neutrophils actively contribute to the stromal–immune landscape that defines PDAC by facilitating immune evasion, modifying the extracellular matrix, and promoting tumor cell invasion [214]. While earlier models attempted to classify tumor-associated neutrophils into dichotomous “antitumor” or “protumor” phenotypes, analogous to macrophage polarization, recent high-resolution transcriptomic analyses have revealed a continuum of neutrophil states shaped by local environmental cues. This phenotypic plasticity underscores their adaptability and functional heterogeneity within the TME [215]. Clinically, neutrophil-associated gene signatures, including markers of effector activity, have demonstrated prognostic value independent of traditional staging, supporting the notion that neutrophil-driven programs reflect meaningful differences in immune suppression, tissue remodeling, and therapeutic resistance across PDAC tumors [216].

Neutrophils play an active role in PDAC progression through multiple effector pathways, among which the release of NETs has gained particular attention [217]. In PDAC, high levels of NETs and dense neutrophil infiltration have been consistently associated with worse progression-free and disease-specific survival [218]. Their presence has emerged as an adverse prognostic feature that provides additional predictive value beyond traditional clinical and pathological criteria [218,219]. Patients with minimal neutrophil infiltration or absent intratumoral NETs are more likely to respond to fluoropyrimidine- or gemcitabine-based chemotherapy, suggesting that NET-enriched microenvironments may buffer cytotoxic injury through stromal–immune reprogramming [218]. Systemically, this biology is mirrored by peripheral markers such as the neutrophil-to-lymphocyte ratio (NLR), which correlates with poor clinical outcomes and reflects an overarching inflammatory state driven by neutrophil dominance [220].

At the mechanistic level, NETs contribute to PDAC progression by directly enhancing tumor cell plasticity and migration while simultaneously remodeling the surrounding stroma [103,217,221]. Exposure to NETs has been shown to induce EMT in PDAC cells, promoting invasive behavior and aligning with a model in which NET scaffolds serve as concentrated platforms for bioactive proteases and inflammatory mediators that alter tumor cell phenotype [221,222]. Concurrently, NETs activate pancreatic stellate cells (PSCs), which drive fibrotic remodeling and establish a feedback loop in which the desmoplastic stroma supports tumor expansion and maintains neutrophil recruitment and NET formation [103,223]. Preclinical studies have demonstrated that disrupting NETs—through DNase treatment, inhibition of PAD4, or interference with neutrophil chemotaxis via C X C chemokine receptor 1 and 2 (CXCR1/2) blockade—can delay tumor growth and improve survival in orthotopic PDAC models, supporting a direct causal role for NETs in disease progression [212]. Additional findings from neoadjuvant-treated patient cohorts further link NET abundance to poor therapeutic response. Increased expression of NET markers such as MPO and CitH3 has been observed in non-responders, while experimental PAD4 inhibition reduces NET formation, circulating NET-derived DNA, and tumor invasiveness [212,224]. Together, these insights support a model in which chemotherapy and tumor-derived stressors promote neutrophil activation and NETosis, while NET-rich microenvironments, in turn, reinforce treatment resistance and tumor dissemination, particularly diminishing the efficacy of standard therapies such as gemcitabine and nab-paclitaxel in specific PDAC contexts

### 8.2. ChemoNETosis as a Driver of Resistance in Pancreatic Ductal Adenocarcinoma

In pancreatic ductal adenocarcinoma, gemcitabine-based chemotherapy has been shown to paradoxically provoke an inflammatory reprogramming of tumor cells that fosters neutrophil infiltration and primes them for NET release [224]. Rather than acting directly on neutrophils, gemcitabine appears to exert this effect indirectly by promoting the secretion of neutrophil-activating cytokines and chemokines from PDAC cells, including CXCL2, CXCL8 also known as interleukin 8 (IL-8), and IL-1β [224]. This secretory shift is thought to be redox-driven, with increased ROS production—partly mediated by nicotinamide adenine dinucleotide phosphate (NADPH) oxidase—activating transcriptional regulators such as nuclear factor kappa B (NF-κB) and signal transducer and activator of transcription 3 (STAT3) [89,225]. These transcriptional changes enhance CXCL8 expression, creating a chemokine gradient that not only attracts neutrophils into the TME but also primes them for NETosis in response to local cues [224].

Within this inflammatory circuit, CXCL8 functions as a central driver by engaging CXCR1 and CXCR2 on neutrophils. This engagement not only directs chemotaxis but also sensitizes neutrophils to undergo NET release [224]. Experimental data have shown that CXCL8 alone is sufficient to induce NETosis in vitro, and tumor CXCL8 levels have been correlated with NET abundance in several cancers, including PDAC [212]. These findings support the biological relevance of this axis in vivo. Notably, pharmacologic antagonists of CXCR1/2 such as reparixin and navarixin can suppress NET formation, particularly when CXCR2 is targeted, reflecting its stronger role in neutrophil activation under inflammatory conditions. These agents offer a potential therapeutic strategy for modulating NET-driven resistance in PDAC by disrupting the tumor–neutrophil interaction at the level of chemokine receptor signaling [224].

Once NETs accumulate within the TME, they can counteract the cytotoxic effects of chemotherapy by supporting tumor cell survival [212]. NET exposure has been associated with reduced intercellular adhesion and attenuated apoptotic response to gemcitabine [224]. Mechanistically, PDAC cells exposed to NETs exhibit a shift in apoptotic balance, with increased expression of anti-apoptotic B-cell lymphoma-extra large (Bcl-xL) and decreased levels of pro-apoptotic Bcl-2-associated X protein (BAX). These changes are accompanied by enhanced activation of extracellular signal-regulated kinases 1 and 2 (ERK1/2), which are key regulators of survival pathways and mitochondrial integrity [224]. This interplay forms a feedback loop where chemotherapy induces an inflammatory tumor secretome that triggers NETosis, and the resulting NET-rich milieu stabilizes pro-survival signaling in tumor cells, thereby blunting the intended cytotoxic response.

A more defined resistance mechanism has been identified in the context of gemcitabine combined with nanoparticle albumin-bound paclitaxel (GnP), involving the G protein-coupled receptor class C group 5 member A (GPRC5A) [212]. In this model, exposure to GnP promotes a transcriptional phenotype in PDAC cells marked by elevated GPRC5A expression and a corresponding surge in CXCL8 production [212]. This chemokine release enhances neutrophil infiltration and NET formation within the tumor. Importantly, this phenomenon does not occur when neutrophils are exposed to GnP in isolation, but rather requires co-culture with tumor cells, reinforcing the idea that chemoNETosis is a tumor-instructed process [212]. Experimental inhibition or genetic silencing of GPRC5A leads to reduced CXCL8 secretion and diminished NETosis, supporting GPRC5A as a modifiable driver of chemoresistance in the GnP setting [212].

**Figure 3:**
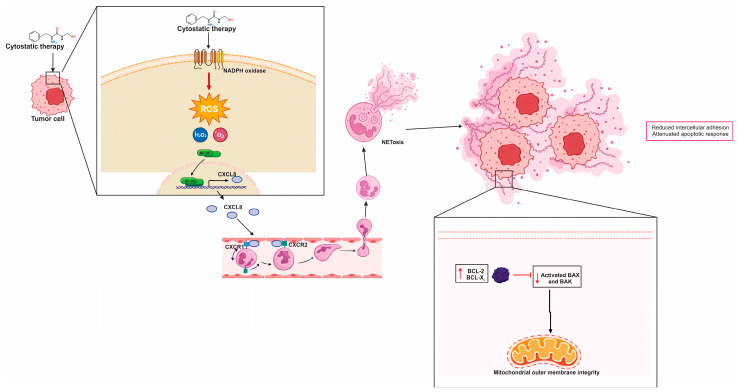
ChemoNETosis in pancreatic ductal adenocarcinoma: gemcitabine-driven oxidative stress promotes CXCL8-dependent neutrophil recruitment and NET release, which in turn dampens apoptosis and supports tumor cell survival. The schematic illustrates an indirect, tumor-instructed mechanism by which gemcitabine-based therapy can promote NETosis within the PDAC microenvironment. Cytostatic treatment increases reactive oxygen species (ROS) in tumor cells—partly via NADPH oxidase—thereby activating redox-responsive transcriptional regulators (e.g., NF-κB and STAT3) that enhance CXCL8 production. Secreted CXCL8 establishes a chemotactic gradient and engages CXCR1/CXCR2 on circulating neutrophils, promoting their recruitment into the tumor and priming them for NETosis in response to local inflammatory cues. NETs deposited in the tumor bed then reshape tumor cell behavior by reducing intercellular adhesion and attenuating the apoptotic response to chemotherapy. At the level of mitochondrial apoptosis control, NET exposure is depicted as shifting the balance toward survival, with increased anti-apoptotic BCL-2/BCL-xL signaling and reduced activation of BAX/BAK, thereby preserving mitochondrial outer membrane integrity and limiting downstream apoptotic execution. Overall, the figure emphasizes a feed-forward loop in which chemotherapy-induced inflammatory reprogramming promotes NET formation, and NETs subsequently reinforce chemoresistant, pro-survival states in PDAC cells.

Further downstream, inflammasome components such as nucleotide-binding domain leucine-rich repeat-containing protein 3 (NLRP3) have been implicated in amplifying NET production and reinforcing treatment failure [212,226]. CXCL8 enhances NLRP3 signaling in neutrophils, facilitating NETosis and the release of cfDNA, which can in turn stimulate tumor cell proliferation and migration [212]. This creates a layered feed-forward loop: GPRC5A-driven CXCL8 secretion attracts neutrophils, NLRP3 activation supports NET and cfDNA release, and these neutrophil-derived products reinforce tumor aggressiveness while dampening chemotherapy response. From a therapeutic perspective, this suggests that dismantling the GPRC5A–NF-κB–CXCL8–NLRP3–NET/cfDNA axis may restore treatment sensitivity by uncoupling the reciprocal tumor–neutrophil signals that sustain resistance to GnP in PDAC [212]. These interactions are summarized in Figure 3, which presents chemoNETosis in PDAC as a tumor-instructed inflammatory circuit that is initiated by gemcitabine-driven oxidative stress and culminates in NET-dependent survival signaling.

In summary, pancreatic ductal adenocarcinoma remains a paradigmatic lethal malignancy in which late presentation, profound desmoplasia, aberrant vasculature, and multilayered immunosuppression converge to limit drug delivery and blunt durable responses to gemcitabine-based regimens, including gemcitabine plus nab-paclitaxel. Within this hostile microenvironment, neutrophils emerge as dynamic architects of immune–stromal remodeling, and NET formation is increasingly linked to adverse biology, inferior progression-free and disease-specific survival, and reduced responsiveness to fluoropyrimidine- or gemcitabine-based therapy, with systemic correlates such as an elevated neutrophil-to-lymphocyte ratio reflecting neutrophil-dominant inflammation. Mechanistically, NETs can amplify PDAC plasticity and invasiveness by promoting EMT programs and by activating pancreatic stellate cells, thereby reinforcing fibrotic remodeling and sustaining a feed-forward loop of neutrophil recruitment and NETosis; importantly, preclinical disruption of NETs (DNase, PAD4 inhibition, or CXCR1/2 blockade) delays tumor growth and improves survival, supporting a causal contribution. In the chemoNETosis framework, cytotoxic injury instructs tumor cells to adopt an inflammatory secretory phenotype—often via ROS/NADPH oxidase-linked activation of NF-κB and STAT3—that elevates CXCL8 (IL-8), CXCL2, and IL-1β, recruits neutrophils, and primes NET release through CXCR1/2 signaling; once established, NET-rich niches can attenuate gemcitabine-induced apoptosis by shifting the Bcl-xL/BAX balance and activating ERK1/2 survival pathways. A more genetically anchored resistance circuit has been described under gemcitabine + nab-paclitaxel (GnP), where treatment selects for a GPRC5A-high tumor state that escalates CXCL8 output and tumor-instructed NETosis, with downstream amplification through NLRP3 signaling and NET/cfDNA-mediated pro-tumor effects—together defining a tractable GPRC5A–NF-κB–CXCL8–NLRP3–NET/cfDNA axis for combination strategies. Finally, the field’s near-term translational bottlenecks include non-standardized definitions and assays for chemoNETosis, limited causal and spatial evidence in human specimens, incomplete understanding of NET cargo as a functional determinant, and the need to balance NET-directed interventions against infection risk—priorities that argue for harmonized biomarker panels, longitudinal/spatial sampling, and rationally timed combination trials. Key mechanistic checkpoints, biomarker candidates, and clinically actionable implications are summarized in Table 3.

Taken together, the available evidence indicates that chemoNETosis operates across breast, colorectal, and pancreatic cancer through several shared biological themes, including therapy-driven inflammatory signaling, NET-mediated remodeling of the tumor microenvironment, enhancement of tumor-cell survival and dissemination, and promotion of resistance-associated programs. At the same time, important differences remain evident in the dominant upstream triggers, the molecular mediators involved, and the specific downstream consequences observed in each tumor type, reflecting the context-dependent nature of NET biology across distinct malignancies. This comparative perspective not only highlights the broader relevance of chemoNETosis in solid tumors, but also underscores the need to address the unresolved mechanistic and translational questions discussed in the following section.

## 9. Knowledge Gaps and Future Directions

One of the most pressing challenges in advancing the concept of chemotherapy-induced neutrophil extracellular trap formation, or chemoNETosis, lies in the lack of standardized definitions and reliable measurement tools. Although this review frames chemoNETosis as a therapy-driven inflammatory response that reshapes the tumor microenvironment and triggers NET release with consequences for treatment efficacy, current studies use a wide variety of readouts and inconsistent biomarkers. Moving forward, it is essential to establish harmonized criteria that distinguish chemoNETosis from baseline NETosis, along with standardized protocols for sample collection—including plasma versus serum, primary versus metastatic sites, and timing relative to chemotherapy infusion. Ideally, multi-parameter panels should integrate both structural NET markers such as CitH3 and NE-DNA complexes, and functional metrics like NET degradability, protease activity, and thrombotic potential. The field would benefit from coordinated ring trials across laboratories to validate assay performance, enabling cross-cohort comparisons and more robust clinical correlations.

Another major limitation is the incomplete understanding of causality and spatial organization in human disease. Most current data sets demonstrate association rather than causation, leaving critical gaps in the mechanistic sequence that links treatment-induced tumor stress, chemokine signaling, neutrophil recruitment, NET formation, and therapy resistance. Addressing this will require careful spatial and temporal profiling, including pre- and on-treatment tissue sampling from both primary tumors and metastatic lesions. The integration of single-cell and spatial transcriptomic or proteomic technologies could help to pinpoint which tumor or stromal compartments initiate NET-inducing signals and which neutrophil phenotypes mediate downstream effects. Simultaneously, deeper profiling of NET cargo—including enzymes, oxidants, and signaling molecules—is crucial, as NETs are increasingly recognized not as inert DNA scaffolds, but as biologically active platforms that shape immune evasion, vascular remodeling, and epithelial-to-mesenchymal transition.

The prognostic relevance of chemoNETosis across different cancer types also remains underexplored. While this review outlines compelling mechanistic evidence in breast cancer, PDAC, and CRC, it is still unclear whether chemoNETosis is a broadly applicable resistance mechanism or a phenomenon limited to specific tumor contexts. To resolve this, future studies should validate NET-related signatures as dynamic biomarkers in a wider range of malignancies, both solid and hematologic. Such efforts must use standardized clinical endpoints, including response rates, progression-free survival, treatment-related toxicity, and thrombotic events. Importantly, biomarker studies must disentangle general inflammation from NET-specific biology by showing that treatment-evoked NET formation adds predictive value beyond traditional markers such as circulating neutrophil counts, C-reactive protein (CRP), or elevated cytokines.

Therapeutically, the question of how to disrupt NET-mediated resistance without impairing host defense remains central. Preclinical studies suggest that targeting PAD4, dismantling NETs using DNase, or blocking upstream inflammatory pathways such as the CXCL1 and CXCL5–CXCR2 axis or IL-1β signaling may restore treatment sensitivity. The next logical step is to design rational combination regimens that incorporate NET-targeting strategies with chemotherapy. These may include agents that prevent NET formation, those that degrade existing NETs timed to coincide with peak release, or those that block downstream consequences of NET activity such as epithelial–mesenchymal transition-related signaling. However, since neutrophils are essential for protecting against infection, clinical trials must incorporate immune monitoring and risk stratification, especially to determine whether NET inhibition can also mitigate chemotherapy-related toxicity, as suggested by vascular injury patterns observed in breast cancer models.

Lastly, a nuanced view of chemoNETosis is required, recognizing that its impact may vary based on tumor genetics and metabolic state. The evidence suggests that in some contexts, NETs may contribute to antitumor responses, rather than solely driving resistance. Future research should focus on identifying the tumor-intrinsic factors—such as oncogenic mutations, redox status, and metabolic profiles—that influence the function of NETs. Additionally, it is important to extend prognostic and predictive frameworks to tumor types beyond those currently studied, mapping where NETs mark immune engagement, resistance, or therapeutic opportunity. Ultimately, the goal is to create clinical decision algorithms that define a patient’s chemoNETosis state—based on biomarkers, spatial localization, and timing—and guide personalized interventions, whether that involves blocking neutrophil recruitment, inhibiting PAD4, applying DNase, or refraining from NET inhibition when the inflammatory response appears to support treatment efficacy.

## 10. Conclusions

ChemoNETosis can be understood as an inflammatory program initiated by chemotherapy and shaped by tumor-derived signals, where treatment-induced stress reconfigures the cytokine and chemokine environment to attract neutrophils and stimulate the release of NETs within both primary tumors and metastatic niches. In this context, NETs are not simply markers of inflammation, but dynamic extracellular structures that influence therapeutic outcomes by concentrating proteolytic enzymes, reactive species, and signaling mediators that modify the vascular and stromal microenvironment and promote tumor cell plasticity and drug tolerance. This review emphasizes that chemoNETosis is not only biologically significant but also therapeutically targetable, converging on shared mechanistic pathways such as chemokine-guided neutrophil recruitment, inflammasome-linked cytokine signaling, and PAD4-mediated chromatin decondensation. At the same time, its functional effects are context-dependent and may, under certain conditions, support antitumor immunity. Defining the conditions under which NET formation drives resistance versus response remains a critical goal. To move this field forward, efforts should focus on standardizing NET detection, incorporating both circulating and tissue biomarkers into clinical research, and developing combination strategies that selectively disrupt NET formation—such as inhibition of PAD4, enzymatic degradation of NET scaffolds, or targeting upstream chemokine pathways—in order to enhance therapeutic efficacy while minimizing systemic toxicity.

## Figures and Tables

**Table 1:** ChemoNETosis in breast cancer—mechanistic checkpoints, biomarkers, and translational implications.

Process/Node	Core Mechanism	Evidence Context	Clinical Significance/Therapeutic Implication	Key Take-Home Message	References
Neutrophils in the breast TME	Neutrophil phenotypes shift with local cytokines, chemokines, and metabolic stress, enabling either antitumor activity or support of progression and immune suppression	TME-focused observational and mechanistic studies	Encourages patient stratification by inflammatory myeloid signatures when interpreting therapy response	Neutrophil function is context dependent and can become a decisive modifier of treatment efficacy	[134,135,136,137,138]
NETosis as an active driver (not a bystander)	NET formation (suicidal, vital, mitochondrial routes) can reshape macrophage and fibroblast programs and reinforce aggressive tumor behavior	Observational associations and functional models	Positions NET burden as a candidate biomarker and a rational target to complement standard therapy	NETs can actively remodel the microenvironment in biologically aggressive disease	[139,140,144]
NET biomarkers in circulation	CitH3, NE–DNA complexes, and cfDNA are being evaluated as proxies of NET activity and metastatic propensity	Translational biomarker investigations	Potential for minimally invasive monitoring, but requires standardization and subtype-aware interpretation	Circulating NET markers may help track inflammatory resistance programs over time	[139]
ChemoNETosis initiation circuit	Tumor stress releases ATP; surviving cells sense ATP via P2X7, activating NLRP3 and caspase-1 to mature IL-1β, which then drives NETosis in recruited neutrophils	Murine metastatic niche models (especially lung)	Identifies upstream checkpoints (P2X7, NLRP3, IL-1β) that could be therapeutically leveraged without broadly suppressing immunity	Chemotherapy can create a cytokine circuit that indirectly instructs NETosis at metastatic sites	[119,147,148,149]
NET scaffold–TGF-β axis and EMT	NETs concentrate latent TGF-β and promote its activation (αvβ1 integrin binding and MMP-9 processing), triggering SMAD2 signaling and EMT-linked therapy tolerance	Mechanistic studies in metastatic lesions	Supports combining standard chemotherapy with NET-disrupting strategies to reduce EMT-driven persistence	NETs can localize and amplify TGF-β signaling, making resistance a microenvironmental event	[119,149,150,151,152,153,154,155,156]
Toxicity and vascular injury programs	NET-forming neutrophils can contribute to nephrotoxicity; chemotherapy-related NETosis is also linked to endothelial injury, glycocalyx disruption, and a prothrombotic phenotype (including iron/ROS/PAD4-linked pathways)	Preclinical toxicity models and neoadjuvant clinical observations	NET targeting may improve tolerability and perioperative vascular safety while supporting oncologic control	NET inhibition may deliver “two wins”: better efficacy and reduced collateral damage	[119,146,153,154,155,156]

**Table 2:** ChemoNETosis and NET biology in colorectal cancer—dual roles, actionable checkpoints, and clinical meaning.

Program/Context	Core Mechanism	Evidence Context	Clinical Significance/Therapeutic Implication	Key Take-Home Message	References
Neutrophils in the CRC TME	Neutrophil programs are shaped by the inflammatory setting of sporadic, hereditary, and colitis-associated CRC, enabling either tumor restraint or tumor-promoting immune suppression	Human tumor/TME profiling and inflammation-driven CRC concepts; supported by mechanistic literature on myeloid conditioning	Supports patient stratification by inflammatory/myeloid signatures when interpreting prognosis and therapy response	Neutrophils are highly plastic; the local milieu largely determines their net impact	[167,169,170,171,172,173]
NETs as metastatic scaffolds	NETs create DNA–protein meshes that persist in inflamed tissue, concentrate cytokines, and can trap circulating tumor cells to enhance endothelial adhesion, extravasation, and organ seeding (liver/lung)	Detection of NETs in CRC tissues and metastasis-related experimental observations, with translational signals in patients	Rationale for NET-disrupting approaches to reduce metastatic seeding and inflammation-enabled relapse	NETs can facilitate dissemination by acting as adhesive, cytokine-rich microplatforms	[175,186,196,197,198]
Perioperative NET amplification	Surgical trauma and systemic inflammation can amplify CXCR2-linked neutrophil recruitment and NET formation, creating a permissive niche for residual tumor outgrowth	Preclinical surgical-stress models and clinical correlations after resection (including liver metastasis surgery)	Identifies the perioperative window as a practical timepoint for NET-directed mitigation strategies	The perioperative phase may be a high-risk “NET-permissive” interval for recurrence	[201]
Targeting recruitment and NET formation	Rather than neutrophil depletion, therapeutic focus shifts to IL-8/CXCR1/2-mediated trafficking and PAD4-dependent chromatin remodeling; additional regulators include G-CSF, TGF-β, VEGF, and JAK/STAT-linked inflammatory pathways	Preclinical targeting studies and translational rationale based on neutrophil biology; emerging combination concepts	Enables more selective immunomodulation while preserving host defense, and may improve radiosensitivity/therapy responsiveness	Selective pathway modulation is more feasible than broad neutrophil suppression in CRC	[173,176,177,178,179,180,184]
CRC chemoNETosis with antitumor effect (PIK3CA-mutant setting)	CB-839 plus 5-FU induces ROS and activates NRF2 in tumor cells, increasing IL-8, recruiting neutrophils, and triggering PAD4-dependent NET release within the tumor bed	Murine CRC chemoNETosis model with genotype-specific dependency (PIK3CA-mutant)	Suggests a biomarker-defined subset where NET induction may augment cytotoxic efficacy rather than drive resistance	ChemoNETosis can be beneficial when regimen, redox signaling, and tumor genotype align	[200,201,202]
NET cargo-mediated apoptosis (CTSG axis)	NET-associated CTSG enters CRC cells via RAGE, cleaves 14-3-3 proteins, releases apoptotic constraints, and enables BAX mitochondrial translocation; higher post-treatment NET levels associate with improved PFS	Mechanistic pathway mapping plus translational association with outcomes in treated samples	Shifts attention from NET “quantity” alone to NET “composition,” supporting cargo-aware biomarkers and therapeutic design	NET biology is outcome-defining only in context—cargo and upstream triggers determine whether NETs harm or help	[84,99,199]

**Table 3:** ChemoNETosis in PDAC—mechanistic checkpoints, biomarkers, and translational implications.

Program/Context	Core Mechanism (Humanized Summary)	Evidence Context	Clinical Significance/Therapeutic Implication	Key Take-Home Message	References
Neutrophil enrichment and NET burden as adverse biology	Neutrophils in PDAC adopt diverse, microenvironment-conditioned states; NET-rich tumors align with aggressive behavior and weaker chemotherapy responsiveness	Human correlative studies (intratumoral NET markers; outcome associations) and supportive mechanistic work	Positions neutrophil/NET signatures as prognostic and potentially predictive biomarkers beyond standard staging	NET-rich inflammation is not a bystander in PDAC; it often tracks with poor outcomes and therapy refractoriness	[214,215,219,220]
Systemic inflammatory correlates	Peripheral neutrophil dominance (e.g., elevated NLR) mirrors tumor immune skewing and adverse clinical trajectory	Clinical observational associations	Low-cost, widely available inflammatory indices may support risk stratification and trial enrichment when paired with NET-specific markers	Systemic inflammation can reflect, but not fully define, NET-driven tumor biology—NET-specific assays are needed	[221]
NET-driven stromal and epithelial reprogramming	NET exposure promotes EMT-like plasticity and migration, while activating pancreatic stellate cells to reinforce desmoplasia and sustain neutrophil recruitment	Preclinical mechanistic studies in PDAC models	Supports targeting NET–stroma coupling to weaken the fibrotic, drug-excluding niche and limit invasion	NETs function as bioactive platforms that rewire both tumor cells and stroma toward persistence	[103,219,222,223,224]
NET targeting in PDAC models	Disrupting NETs (DNase), inhibiting PAD4, or blocking CXCR1/2-mediated recruitment reduces NET burden, delays tumor growth, and improves survival	Orthotopic/preclinical PDAC models; signals from neoadjuvant-treated cohorts regarding response	Provides a rationale for combination regimens pairing chemotherapy with NET-directed agents, ideally biomarker-guided	NET inhibition can be therapeutically meaningful when applied to NET-enriched PDAC contexts	[213,225]
Gemcitabine-driven chemoNETosis (inflammatory secretome → NETosis)	Cytotoxic stress induces a tumor secretory shift (CXCL2, CXCL8/IL-8, IL-1β), often linked to ROS/NADPH oxidase and NF-κB/STAT3 activation, recruiting and priming neutrophils via CXCR1/2 for NET release	Mechanistic and co-culture/conditioning models supporting tumor-instructed neutrophil activation	Highlights upstream nodes (ROS signaling, NF-κB/STAT3, CXCL8–CXCR1/2) as druggable levers to reduce NET-driven resistance	In PDAC, chemoNETosis is largely tumor-instructed rather than a direct drug effect on neutrophils	[89,225,226]
NET-mediated blunting of cytotoxicity and GnP-specific resistance axis	NET-rich niches reduce apoptosis and favor survival signaling (Bcl-xL up, BAX down; ERK1/2 activation); under GnP, GPRC5A elevation boosts CXCL8, promoting NETosis and downstream amplification through NLRP3 with NET/cfDNA pro-tumor effects	Preclinical pathway definition with translational support; emphasis on tumor–neutrophil reciprocity	Suggests multi-node combination targeting (GPRC5A, CXCL8–CXCR1/2, NLRP3, NET/cfDNA) to restore sensitivity and limit aggressive escape	Resistance can be sustained by reciprocal tumor–neutrophil loops; breaking the loop may re-open a therapeutic window	[213]

## Data Availability

No new data were created or analyzed in this study.

## Abbreviations

The following abbreviations are used in this manuscript:

5-FU	5-fluorouracil
ATP	Adenosine triphosphate
BAX	Bcl-2-associated X protein
BCL-2	B-cell lymphoma 2
Bcl-xL	B-cell lymphoma-extra large
Bv8	Prokineticin-2
CB-839	Glutaminase inhibitor CB-839
CCDC25	Coiled-coil domain containing protein 25
CDC42	Cell division cycle 42
CD8+	Cluster of differentiation 8 positive
CEACAM1	Carcinoembryonic antigen-related cell adhesion molecule 1
cfDNA	Cell-free DNA
CitH3	Citrullinated histone H3
COPD	Chronic obstructive pulmonary disease
CRP	C-reactive protein
CRC	Colorectal cancer
CSF3R	Colony-stimulating factor 3 receptor
CTCs	Circulating tumor cells
CTLA-4	Cytotoxic T lymphocyte-associated protein 4
CTSG	Cathepsin G
ctDNA	Circulating tumor DNA
CXCL	C X C motif chemokine ligand
CXCL1	C X C motif chemokine ligand 1
CXCL2	C X C motif chemokine ligand 2
CXCL5	C X C motif chemokine ligand 5
CXCL8	C X C motif chemokine ligand 8
CXCL8/IL-8	C X C motif chemokine ligand 8/interleukin 8
CXCR1	C-X-C motif chemokine receptor 1
CXCR1/2	C X C chemokine receptor 1 and 2
CXCR2	C X C chemokine receptor 2
CXCR4	C-X-C motif chemokine receptor 4
DAMP	Damage-associated molecular pattern
DAMPs	Damage-associated molecular patterns
DNase	Deoxyribonuclease
DNase I	Deoxyribonuclease I
ECM	Extracellular matrix
EMT	Epithelial to mesenchymal transition
ER-positive	Estrogen receptor-positive
ERK	Extracellular signal-regulated kinase
ERK1/2	Extracellular signal-regulated kinases 1 and 2
FCGR3B	Fc gamma receptor IIIb
G-CSF	Granulocyte colony-stimulating factor
GM-CSF	Granulocyte macrophage colony-stimulating factor
GnP	Gemcitabine plus nanoparticle albumin-bound paclitaxel
GLOBOCAN	Global Cancer Observatory
GPRC5A	G protein-coupled receptor class C group 5 member A
ICAM1	Intercellular adhesion molecule 1
IL-1β	Interleukin 1 beta
IL-8	Interleukin 8
IL-8/CXCL8	Interleukin 8/C-X-C motif chemokine ligand 8
IL-17A	Interleukin 17A
ILK	Integrin-linked kinase
JAK/STAT	Janus kinase/signal transducer and activator of transcription
JAK/STAT3	Janus kinase/signal transducer and activator of transcription 3
JAK2/STAT3	Janus kinase 2/signal transducer and activator of transcription 3
KRAS	Kirsten rat sarcoma viral oncogene homolog
MAPK	Mitogen-activated protein kinase
mCRC	Metastatic colorectal cancer
MDSCs	Myeloid-derived suppressor cells
MMP-9	Matrix metalloproteinase 9
MPO	Myeloperoxidase
nab-paclitaxel	Nanoparticle albumin-bound paclitaxel
NADPH	Nicotinamide adenine dinucleotide phosphate
N-cadherin	Neural cadherin
NE	Neutrophil elastase
NE–DNA	Neutrophil elastase–DNA complexes
NET	Neutrophil extracellular trap
NETs	Neutrophil extracellular traps
NETosis	Neutrophil extracellular trap formation (NET formation)
NF-κB	Nuclear factor kappa B
NK	Natural killer
NLR	Neutrophil-to-lymphocyte ratio
NLRP3	Nucleotide-binding domain leucine-rich repeat-containing protein 3
NRF2	Nuclear factor erythroid 2-related factor 2
OLR1	Oxidized low-density lipoprotein receptor 1
P2X7	Purinergic P2X7 receptor
PAD4	Peptidylarginine deiminase 4
PD-1	Programmed cell death protein 1
PD-1/PD-L1	Programmed death 1/programmed death ligand 1
PD-L1	Programmed death ligand 1
PDAC	Pancreatic ductal adenocarcinoma
PET-CT	Positron emission tomography combined with computed tomography
PI3K/AKT	Phosphoinositide 3-kinase/protein kinase B
PIK3CA	Phosphatidylinositol 4,5-bisphosphate 3-kinase catalytic subunit alpha
PMNs	Polymorphonuclear leukocytes
PRTN3	Proteinase 3
PSCs	Pancreatic stellate cells
PTGS2	Prostaglandin-endoperoxide synthase 2
RA	Rheumatoid arthritis
RAC1	Ras-related C3 botulinum toxin substrate 1
RAGE	Receptor for advanced glycation end products
ROS	Reactive oxygen species
Slc11a1	Solute carrier family 11 member 1
SLE	Systemic lupus erythematosus
SMAD2	SMAD family member 2
STAT3	Signal transducer and activator of transcription 3
TANs	Tumor-associated neutrophils
TGF-β	Transforming growth factor beta
TME	Tumor microenvironment
TLRs	Toll-like receptors
TNBC	Triple-negative breast cancer
TNF-α	Tumor necrosis factor alpha
TP53	Tumor protein p53
Tregs	Regulatory T cells
VEGF	Vascular endothelial growth factor
VEGFA	Vascular endothelial growth factor A
vWF	von Willebrand factor

## References

[1] Bray F., Laversanne M., Sung H., Ferlay J., Siegel R.L., Soerjomataram I., Jemal A. (2024). Global cancer statistics 2022: GLOBOCAN estimates of incidence and mortality worldwide for 36 cancers in 185 countries. CA Cancer J. Clin..

[2] Meattini I., Becherini C., Caini S., Coles C.E., Cortes J., Curigliano G., de Azambuja E., Isacke C.M., Harbeck N., Kaidar-Person O. (2024). International multidisciplinary consensus on the integration of radiotherapy with new systemic treatments for breast cancer: European Society for Radiotherapy and Oncology (ESTRO)-endorsed recommendations. Lancet Oncol..

[3] Gerstberger S., Jiang Q., Ganesh K. (2023). Metastasis. Cell.

[4] Yan Y., Yuan J., Peng Y., Zhou C., Liu X., Sun L., Song Q. (2025). Bispecific antibodies combined with chemotherapy in solid tumor treatment, the path forward?. Front. Immunol..

[5] Gonçalves A.C., Richiardone E., Jorge J., Polónia B., Xavier C.P.R., Salaroglio I.C., Riganti C., Vasconcelos M.H., Corbet C., Sarmento-Ribeiro A.B. (2021). Impact of cancer metabolism on therapy resistance—Clinical implications. Drug Resist. Updates.

[6] Grant G., Ferrer C.M. (2025). The role of the immune tumor microenvironment in shaping metastatic dissemination, dormancy, and outgrowth. Trends Cell Biol..

[7] Sabit H., Arneth B., Abdel-Ghany S., Madyan E.F., Ghaleb A.H., Selvaraj P., Shin D.M., Bommireddy R., Elhashash A. (2024). Beyond Cancer Cells: How the Tumor Microenvironment Drives Cancer Progression. Cells.

[8] Dhanyamraju P.K., Schell T.D., Amin S., Robertson G.P. (2022). Drug-Tolerant Persister Cells in Cancer Therapy Resistance. Cancer Res..

[9] Iborra F.J., Martí C., Calabuig-Navarro V., Papadopoulos P., Meseguer S., Iborra P.M., García F., Martínez-Lorente A., Almazán F., Calabuig J. (2023). Chemotherapy induces cell plasticity; controlling plasticity increases therapeutic response. Signal Transduct. Target. Ther..

[10] de Bakker T., Maes A., Dragan T., Martinive P., Penninckx S., Van Gestel D. (2024). Strategies to Overcome Intrinsic and Acquired Resistance to Chemoradiotherapy in Head and Neck Cancer. Cells.

[11] Dzobo K., Senthebane D.A., Dandara C. (2023). The Tumor Microenvironment in Tumorigenesis and Therapy Resistance Revisited. Cancers.

[12] He J., Qiu Z., Fan J., Xie X., Sheng Q., Sui X. (2024). Drug tolerant persister cell plasticity in cancer: A revolutionary strategy for more effective anticancer therapies. Signal Transduct. Target. Ther..

[13] Nussinov R., Tsai C.J., Jang H. (2021). Anticancer drug resistance: An update and perspective. Drug Resist. Updates.

[14] Mukherjee N., Sheetz J., Shellman Y.G. (2025). Targeting the BCL2 Family: Advances and Challenges in BH3 Mimetic-Based Therapies. Int. J. Mol. Sci..

[15] Jiang M., Zhang K., Zhang Z., Zeng X., Huang Z., Qin P., Xie Z., Cai X., Ashrafizadeh M., Tian Y. (2025). PI3K/AKT/mTOR Axis in Cancer: From Pathogenesis to Treatment. MedComm.

[16] Shi Z.D., Pang K., Wu Z.X., Dong Y., Hao L., Qin J.X., Wang W., Chen Z.S., Han C.H. (2023). Tumor cell plasticity in targeted therapy-induced resistance: Mechanisms and new strategies. Signal Transduct. Target. Ther..

[17] Polak R., Zhang E.T., Kuo C.J. (2024). Cancer organoids 2.0: Modelling the complexity of the tumour immune microenvironment. Nat. Rev. Cancer.

[18] Huang J., Zhang L., Wan D., Zhou L., Zheng S., Lin S., Qiao Y. (2021). Extracellular matrix and its therapeutic potential for cancer treatment. Signal Transduct. Target. Ther..

[19] Maier-Begandt D., Alonso-Gonzalez N., Klotz L., Erpenbeck L., Jablonska J., Immler R., Hasenberg A., Mueller T.T., Herrero-Cervera A., Aranda-Pardos I. (2024). Neutrophils-biology and diversity. Nephrol. Dial. Transpl..

[20] Kraus R.F., Gruber M.A. (2021). Neutrophils-From Bone Marrow to First-Line Defense of the Innate Immune System. Front. Immunol..

[21] Koenderman L., Vrisekoop N. (2025). Neutrophils in cancer: From biology to therapy. Cell. Mol. Immunol..

[22] Liu S., Wu W., Du Y., Yin H., Chen Q., Yu W., Wang W., Yu J., Liu L., Lou W. (2023). The evolution and heterogeneity of neutrophils in cancers: Origins, subsets, functions, orchestrations and clinical applications. Mol. Cancer.

[23] Zhang J., Gu J., Wang X., Ji C., Yu D., Wang M., Pan J., Santos H.A., Zhang H., Zhang X. (2024). Engineering and Targeting Neutrophils for Cancer Therapy. Adv. Mater..

[24] Que H., Fu Q., Lan T., Tian X., Wei X. (2022). Tumor-associated neutrophils and neutrophil-targeted cancer therapies. Biochim. Biophys. Acta Rev. Cancer.

[25] Ng M.S.F., Kwok I., Tan L., Shi C., Cerezo-Wallis D., Tan Y., Leong K., Calvo G.F., Yang K., Zhang Y. (2024). Deterministic reprogramming of neutrophils within tumors. Science.

[26] Tsioumpekou M., Krijgsman D., Leusen J.H.W., Olofsen P.A. (2023). The Role of Cytokines in Neutrophil Development, Tissue Homing, Function and Plasticity in Health and Disease. Cells.

[27] Eruslanov E., Nefedova Y., Gabrilovich D.I. (2025). The heterogeneity of neutrophils in cancer and its implication for therapeutic targeting. Nat. Immunol..

[28] Li J., Hu J., Yang Y., Zhang H., Liu Y., Fang Y., Qu L., Lin A., Luo P., Jiang A. (2025). Drug resistance in cancer: Molecular mechanisms and emerging treatment strategies. Mol. Biomed..

[29] Silvestre-Roig C., Kalafati L., Chavakis T. (2024). Neutrophils are shaped by the tumor microenvironment: Novel possibilities for targeting neutrophils in cancer. Signal Transduct. Target. Ther..

[30] Wu Y., Ma J., Yang X., Nan F., Zhang T., Ji S., Rao D., Feng H., Gao K., Gu X. (2024). Neutrophil profiling illuminates anti-tumor antigen-presenting potency. Cell.

[31] Salcher S., Sturm G., Horvath L., Untergasser G., Kuempers C., Fotakis G., Panizzolo E., Martowicz A., Trebo M., Pall G. (2022). High-resolution single-cell atlas reveals diversity and plasticity of tissue-resident neutrophils in non-small cell lung cancer. Cancer Cell.

[32] Zhou Y., Shen G., Zhou X., Li J. (2025). Therapeutic potential of tumor-associated neutrophils: Dual role and phenotypic plasticity. Signal Transduct. Target. Ther..

[33] Tao L., Li G., Tao M., Xiu D., Yuan C., Ma Z., Jiang B. (2020). Tumor associated neutrophils promote the metastasis of pancreatic ductal adenocarcinoma. Cancer Biol. Ther..

[34] Huang X., Nepovimova E., Adam V., Sivak L., Heger Z., Valko M., Wu Q., Kuca K. (2024). Neutrophils in Cancer immunotherapy: Friends or foes?. Mol. Cancer.

[35] Bird L. (2024). Neutrophils become pro-angiogenic in tumours. Nat. Rev. Immunol..

[36] Buszka K., Dompe C., Derwich K., Pieścikowska I., Nowicki M., Budna-Tukan J. (2025). Dual Nature of Neutrophil Extracellular Traps (NETs)—From Cancer’s Ally to Therapeutic Target. Cells.

[37] de Visser K.E., Joyce J.A. (2023). The evolving tumor microenvironment: From cancer initiation to metastatic outgrowth. Cancer Cell.

[38] Chu C., Wang X., Yang C., Chen F., Shi L., Xu W., Wang K., Liu B., Wang C., Sun D. (2023). Neutrophil extracellular traps drive intestinal microvascular endothelial ferroptosis by impairing Fundc1-dependent mitophagy. Redox Biol..

[39] Cristinziano L., Modestino L., Antonelli A., Marone G., Simon H.U., Varricchi G., Galdiero M.R. (2022). Neutrophil extracellular traps in cancer. Semin. Cancer Biol..

[40] Wahnou H., El Kebbaj R., Hba S., Ouadghiri Z., El Faqer O., Pinon A., Liagre B., Limami Y., Duval R.E. (2025). Neutrophils and Neutrophil-Based Drug Delivery Systems in Anti-Cancer Therapy. Cancers.

[41] Koc D.C., Mănescu I.B., Mănescu M., Dobreanu M. (2024). A Review of the Prognostic Significance of Neutrophil-to-Lymphocyte Ratio in Nonhematologic Malignancies. Diagnostics.

[42] Yang J.J., Hu Z.G., Shi W.X., Deng T., He S.Q., Yuan S.G. (2015). Prognostic significance of neutrophil to lymphocyte ratio in pancreatic cancer: A meta-analysis. World J. Gastroenterol..

[43] Zandi A., Qian A., Othman M. (2025). Optimal Cutoff for the Neutrophil-to-Lymphocyte Ratio as a Tool for Pre-chemotherapy Prognosis Stratification of Breast Cancer Patients. Cureus.

[44] Jaillon S., Ponzetta A., Di Mitri D., Santoni A., Bonecchi R., Mantovani A. (2020). Neutrophil diversity and plasticity in tumour progression and therapy. Nat. Rev. Cancer.

[45] Korbecki J., Bosiacki M., Pilarczyk M., Kot M., Defort P., Walaszek I., Chlubek D., Baranowska-Bosiacka I. (2025). The CXCL1-CXCR2 Axis as a Component of Therapy Resistance, a Source of Side Effects in Cancer Treatment, and a Therapeutic Target. Cancers.

[46] Hou R., Wu X., Wang C., Fan H., Zhang Y., Wu H., Wang H., Ding J., Jiang H., Xu J. (2025). Tumor-associated neutrophils: Critical regulators in cancer progression and therapeutic resistance (Review). Int. J. Oncol..

[47] Gibellini L., Borella R., Santacroce E., Serattini E., Boraldi F., Quaglino D., Aramini B., De Biasi S., Cossarizza A. (2023). Circulating and Tumor-Associated Neutrophils in the Era of Immune Checkpoint Inhibitors: Dynamics, Phenotypes, Metabolism, and Functions. Cancers.

[48] Meng Y., Ye F., Nie P., Zhao Q., An L., Wang W., Qu S., Shen Z., Cao Z., Zhang X. (2023). Immunosuppressive CD10^+^ALPL^+^ neutrophils promote resistance to anti-PD-1 therapy in HCC by mediating irreversible exhaustion of T cells. J. Hepatol..

[49] Zhang Q., Liu S., Wang H., Xiao K., Lu J., Chen S., Huang M., Xie R., Lin T., Chen X. (2023). ETV4 Mediated Tumor-Associated Neutrophil Infiltration Facilitates Lymphangiogenesis and Lymphatic Metastasis of Bladder Cancer. Adv. Sci..

[50] Tang D., Zhang D., Heng Y., Zhu X.K., Lin H.Q., Zhou J., Tao L., Lu L.M. (2022). Tumor-Infiltrating PD-L1+ Neutrophils Induced by GM-CSF Suppress T Cell Function in Laryngeal Squamous Cell Carcinoma and Predict Unfavorable Prognosis. J. Inflamm. Res..

[51] Wang T.T., Zhao Y.L., Peng L.S., Chen N., Chen W., Lv Y.P., Mao F.Y., Zhang J.Y., Cheng P., Teng Y.S. (2017). Tumour-activated neutrophils in gastric cancer foster immune suppression and disease progression through GM-CSF-PD-L1 pathway. Gut.

[52] Michaeli J., Shaul M.E., Mishalian I., Hovav A.H., Levy L., Zolotriov L., Granot Z., Fridlender Z.G. (2017). Tumor-associated neutrophils induce apoptosis of non-activated CD8 T-cells in a TNFα and NO-dependent mechanism, promoting a tumor-supportive environment. Oncoimmunology.

[53] Amorós-Pérez B., Rivas-Pardo B., Gómez Del Moral M., Subiza J.L., Martínez-Naves E. (2024). State of the Art in CAR-T Cell Therapy for Solid Tumors: Is There a Sweeter Future?. Cells.

[54] Pan K., Farrukh H., Chittepu V., Xu H., Pan C.X., Zhu Z. (2022). CAR race to cancer immunotherapy: From CAR T, CAR NK to CAR macrophage therapy. J. Exp. Clin. Cancer Res..

[55] Haist M., Stege H., Grabbe S., Bros M. (2021). The Functional Crosstalk between Myeloid-Derived Suppressor Cells and Regulatory T Cells within the Immunosuppressive Tumor Microenvironment. Cancers.

[56] The Lancet Oncology (2021). CAR T-cell therapy for solid tumours. Lancet. Oncol..

[57] Naish E., Wood A.J., Stewart A.P., Routledge M., Morris A.C., Chilvers E.R., Lodge K.M. (2023). The formation and function of the neutrophil phagosome. Immunol. Rev..

[58] Gierlikowska B., Stachura A., Gierlikowski W., Demkow U. (2021). Phagocytosis, Degranulation and Extracellular Traps Release by Neutrophils-The Current Knowledge, Pharmacological Modulation and Future Prospects. Front. Pharmacol..

[59] Eichelberger K.R., Goldman W.E. (2020). Manipulating neutrophil degranulation as a bacterial virulence strategy. PLoS Pathog..

[60] Yang L., Shi F., Cao F., Wang L., She J., He B., Xu X., Kong L., Cai B. (2025). Neutrophils in Tissue Injury and Repair: Molecular Mechanisms and Therapeutic Targets. MedComm.

[61] Wang H., Kim S.J., Lei Y., Wang S., Wang H., Huang H., Zhang H., Tsung A. (2024). Neutrophil extracellular traps in homeostasis and disease. Signal Transduct. Target. Ther..

[62] Liu M.-L., Lyu X., Werth V.P. (2022). Recent progress in the mechanistic understanding of NET formation in neutrophils. FEBS J..

[63] Schoen J., Euler M., Schauer C., Schett G., Herrmann M., Knopf J., Yaykasli K.O. (2022). Neutrophils’ Extracellular Trap Mechanisms: From Physiology to Pathology. Int. J. Mol. Sci..

[64] Papayannopoulos V. (2018). Neutrophil extracellular traps in immunity and disease. Nat. Rev. Immunol..

[65] Boeltz S., Amini P., Anders H.J., Andrade F., Bilyy R., Chatfield S., Cichon I., Clancy D.M., Desai J., Dumych T. (2019). To NET or not to NET:current opinions and state of the science regarding the formation of neutrophil extracellular traps. Cell Death Differ..

[66] Brambilla M., Zanichelli A., Cancila V., Colombo M.P., Chiodoni C., Sangaletti S. (2025). Neutrophil extracellular traps in cancer: Immune modulation, therapy resistance, and the dilemma of targeting. Cell Death Dis..

[67] Damascena H.L., Silveira W.A.A., Castro M.S., Fontes W. (2022). Neutrophil Activated by the Famous and Potent PMA (Phorbol Myristate Acetate). Cells.

[68] Wang Y., Du C., Zhang Y., Zhu L. (2024). Composition and Function of Neutrophil Extracellular Traps. Biomolecules.

[69] Papayannopoulos V., Metzler K.D., Hakkim A., Zychlinsky A. (2010). Neutrophil elastase and myeloperoxidase regulate the formation of neutrophil extracellular traps. J. Cell Biol..

[70] Burn G.L., Raisch T., Tacke S., Winkler M., Prumbaum D., Thee S., Gimber N., Raunser S., Zychlinsky A. (2025). Myeloperoxidase transforms chromatin into neutrophil extracellular traps. Nature.

[71] Shahzad A., Ni Y., Yang Y., Liu W., Teng Z., Bai H., Liu X., Sun Y., Xia J., Cui K. (2025). Neutrophil Extracellular Traps (NETs) in health and disease. Mol. Biomed..

[72] Szymańska Z., Staniewski A., Karpiński M., Zalewska K., Kalus O., Gramala Z., Maćkowiak J., Mertowski S., Filipiak K.J., Rahnama-Hezavah M. (2025). The Role of Neutrophil Extracellular Networks in Cardiovascular Pathology. Cells.

[73] Tang R., Yin J., Qin Z., Zhang M., Jia X. (2025). NETs: A new target for autoimmune disease. Front. Immunol..

[74] Pérez-Olivares L., Soehnlein O. (2021). Contemporary Lifestyle and Neutrophil Extracellular Traps: An Emerging Link in Atherosclerosis Disease. Cells.

[75] Trivedi A., Khan M.A., Bade G., Talwar A. (2021). Orchestration of Neutrophil Extracellular Traps (Nets), a Unique Innate Immune Function during Chronic Obstructive Pulmonary Disease (COPD) Development. Biomedicines.

[76] Chen J., Wang T., Li X., Gao L., Wang K., Cheng M., Zeng Z., Chen L., Shen Y., Wen F. (2024). DNA of neutrophil extracellular traps promote NF-κB-dependent autoimmunity via cGAS/TLR9 in chronic obstructive pulmonary disease. Signal Transduct. Target. Ther..

[77] Morawiec M.L., Kubina R., Jabłońska E., Ratajczak-Wrona W., Stępień S., Gołębski M., Mielczarek-Palacz A. (2025). NETs—As predictors and targets of supportive therapy for cancer treatment. Front. Immunol..

[78] Zhang H., Wang Y., Onuma A., He J., Wang H., Xia Y., Lal R., Cheng X., Kasumova G., Hu Z. (2021). Neutrophils Extracellular Traps Inhibition Improves PD-1 Blockade Immunotherapy in Colorectal Cancer. Cancers.

[79] Sun X., Gui Y., Yang T., Chen L., Zhang Y., Yan L., Chen W., Wang B. (2024). PD-L1^+^ neutrophils induced NETs in malignant ascites is a potential biomarker in HCC. Cancer Immunol. Immunother..

[80] Deryugina E., Carré A., Ardi V., Muramatsu T., Schmidt J., Pham C., Quigley J.P. (2020). Neutrophil Elastase Facilitates Tumor Cell Intravasation and Early Metastatic Events. iScience.

[81] Albrengues J., Shields M.A., Ng D., Park C.G., Ambrico A., Poindexter M.E., Upadhyay P., Uyeminami D.L., Pommier A., Küttner V. (2018). Neutrophil extracellular traps produced during inflammation awaken dormant cancer cells in mice. Science.

[82] Demkow U. (2021). Neutrophil Extracellular Traps (NETs) in Cancer Invasion, Evasion and Metastasis. Cancers.

[83] Tanigawa K., Kiriya M., Hayashi Y., Shinden Y., Kijima Y., Natsugoe S., Sumimoto T., Morimoto-Kamata R., Yui S., Hama K. (2022). Cathepsin G-induced malignant progression of MCF-7 cells involves suppression of PAF signaling through induced expression of PAFAH1B2. Biochim. Biophys. Acta Mol. Cell Biol. Lipids.

[84] Zhang H., Wang Y., Qu M., Li W., Wu D., Cata J.P., Miao C. (2023). Neutrophil, neutrophil extracellular traps and endothelial cell dysfunction in sepsis. Clin. Transl. Med..

[85] Li H., Zhang Y., Lin J., Zeng J., Liang X., Xu L., Li J., Zhong X., Liu X., Liu Z. (2026). Tumor-derived neutrophil extracellular trap-associated DNA impairs treatment efficacy in breast cancer via CCDC25-dependent epithelial-mesenchymal transition. J. Clin. Investig..

[86] Mousset A., Albrengues J. (2024). NETs unleashed: Neutrophil extracellular traps boost chemotherapy against colorectal cancer. J. Clin. Investig..

[87] Zhang J., Miao C., Zhang H. (2025). Targeting neutrophil extracellular traps in cancer progression and metastasis. Theranostics.

[88] Ricciuti J., Liu Q., Khan A., Joseph J.M., Veuskens B., Giridharan T., Suzuki S., Emmons T., Yaffe M., Kuijpers T.W. (2025). Prognostic significance of serum complement activation, neutrophil extracellular traps and extracellular DNA in newly diagnosed epithelial ovarian cancer. Gynecol. Oncol..

[89] Nigam M., Punia B., Dimri D.B., Mishra A.P., Radu A.F., Bungau G. (2025). Reactive Oxygen Species: A Double-Edged Sword in the Modulation of Cancer Signaling Pathway Dynamics. Cells.

[90] Li J., Xia Y., Sun B., Zheng N., Li Y., Pang X., Yang F., Zhao X., Ji Z., Yu H. (2023). Neutrophil extracellular traps induced by the hypoxic microenvironment in gastric cancer augment tumour growth. Cell Commun. Signal..

[91] Wang X.R., Zhou X.H., Sun X.T., Shen Y.Q., Wu Y.Y., Wu C.D., Zhu F.J., Wei Y.T., Chen J.P., Chen J. (2024). Tumour cell-released autophagosomes promote lung metastasis by upregulating PD-L1 expression in pulmonary vascular endothelial cells in breast cancer. Cell. Oncol..

[92] Zhong W., Wang Q., Shen X., Lv Y., Sun L., An R., Zhu H., Cai H., Chen G., Liu A. (2023). Neutrophil Extracellular Trap is Surrogate Biomarker for Prognosis and Response to Neoadjuvant Therapy in Locally Advanced Rectal Cancer. J. Inflamm. Res..

[93] Xie R., Shang B., Shi H., Bi X., Song Y., Qu W., Bai H., Hu L., Wu J., Cui H. (2023). Neutrophil extracellular traps in relationship to efficacy of systemic therapy for metastatic renal cell carcinoma. Cancer Med..

[94] Xing L., Wu S., Xue S., Li X. (2025). A Novel Neutrophil Extracellular Trap Signature Predicts Patient Chemotherapy Resistance and Prognosis in Lung Adenocarcinoma. Mol. Biotechnol..

[95] Bai Y., Gao Y., Xu H., Gao F., Sun J., Bao P., Zhao J., Jiang T. (2025). Neutrophil extracellular trap-related signature predicts the prognosis and immunotherapy outcome of lung adenocarcinoma. Genomics.

[96] Wang C., Chen J., Song Z., Lv K., Dong H., Liang L., Zhang J., Liu H., Li J., Yang X. (2026). Neutrophil extracellular traps as biomarkers for predicting prognosis and chemotherapy response in colorectal cancer. Sci. Rep..

[97] Baron S., Binenbaum Y., Maman R., Fidel V., Shusterman A., Vaisman D., Sher O., Manisterski M., Shukrun R., Rössig C. (2025). Neutrophil extracellular traps are associated with poor response to neoadjuvant therapy and poor survival in pediatric osteosarcoma. Front. Oncol..

[98] Shahzad M.H., Feng L., Su X., Brassard A., Dhoparee-Doomah I., Ferri L.E., Spicer J.D., Cools-Lartigue J.J. (2022). Neutrophil Extracellular Traps in Cancer Therapy Resistance. Cancers.

[99] Saw P.E., Chen J., Song E. (2023). ChemoNETosis: A road to tumor therapeutic resistance. Cancer Cell.

[100] Jiang Z.Z., Peng Z.P., Liu X.C., Guo H.F., Zhou M.M., Jiang D., Ning W.R., Huang Y.F., Zheng L., Wu Y. (2022). Neutrophil extracellular traps induce tumor metastasis through dual effects on cancer and endothelial cells. Oncoimmunology.

[101] Shang B., Cui H., Xie R., Wu J., Shi H., Bi X., Feng L., Shou J. (2023). Neutrophil extracellular traps primed intercellular communication in cancer progression as a promising therapeutic target. Biomark. Res..

[102] Niemann B., Ivey A., Hopen Q., Dakhlallah D., Brundage K., Mihalik N., Eubank T., Boone B.A. (2025). Neutrophil extracellular trap inhibition revitalizes PDAC immunotherapy responsiveness via reduced fibrosis and TCF1^+^CD8^+^ progenitor T-cell expansion. Oncoimmunology.

[103] Miller-Ocuin J.L., Liang X., Boone B.A., Doerfler W.R., Singhi A.D., Tang D., Kang R., Lotze M.T., Zeh H.J. (2019). DNA released from neutrophil extracellular traps (NETs) activates pancreatic stellate cells and enhances pancreatic tumor growth. Oncoimmunology.

[104] Yoshimoto M., Kagawa S., Kajioka H., Taniguchi A., Kuroda S., Kikuchi S., Kakiuchi Y., Yagi T., Nogi S., Teraishi F. (2023). Dual antiplatelet therapy inhibits neutrophil extracellular traps to reduce liver micrometastases of intrahepatic cholangiocarcinoma. Cancer Lett..

[105] Ren J., He J., Zhang H., Xia Y., Hu Z., Loughran P., Billiar T., Huang H., Tsung A. (2021). Platelet TLR4-ERK5 Axis Facilitates NET-Mediated Capturing of Circulating Tumor Cells and Distant Metastasis after Surgical Stress. Cancer Res..

[106] Yu S., Liu J., Yan N. (2022). Endothelial Dysfunction Induced by Extracellular Neutrophil Traps Plays Important Role in the Occurrence and Treatment of Extracellular Neutrophil Traps-Related Disease. Int. J. Mol. Sci..

[107] Ning Y., Chen Y., Tian T., Gao X., Liu X., Wang J., Chu H., Zhao C., Yang Y., Lei K. (2024). S100A7 orchestrates neutrophil chemotaxis and drives neutrophil extracellular traps (NETs) formation to facilitate lymph node metastasis in cervical cancer patients. Cancer Lett..

[108] Feng C., Li Y., Tai Y., Zhang W., Wang H., Lian S., Jin-Si-Han E.E., Liu Y., Li X., Chen Q. (2023). A neutrophil extracellular traps-related classification predicts prognosis and response to immunotherapy in colon cancer. Sci. Rep..

[109] Liang Y., Wu G., Tan J., Xiao X., Yang L., Saw P.E. (2024). Targeting NETosis: Nature’s alarm system in cancer progression. Cancer Drug Resist..

[110] Santana P.T., de Lima I.S., Silva E.S.K.C.D., Barbosa P.H.S., de Souza H.S.P. (2024). Persistent Activation of the P2X7 Receptor Underlies Chronic Inflammation and Carcinogenic Changes in the Intestine. Int. J. Mol. Sci..

[111] Kaltenmeier C., Yazdani H.O., Morder K., Geller D.A., Simmons R.L., Tohme S. (2021). Neutrophil Extracellular Traps Promote T Cell Exhaustion in the Tumor Microenvironment. Front. Immunol..

[112] Pu D., Yin L., Zhai X., Wang R., Huang L., Wu Q., Zhu L., Zhou Y., Zhou Q., Li L. (2023). The shadows hang over immunotherapy-neutrophil extracellular traps in cancer. Sci. China Life Sci..

[113] Teijeira Á., Garasa S., Gato M., Alfaro C., Migueliz I., Cirella A., de Andrea C., Ochoa M.C., Otano I., Etxeberria I. (2020). CXCR1 and CXCR2 Chemokine Receptor Agonists Produced by Tumors Induce Neutrophil Extracellular Traps that Interfere with Immune Cytotoxicity. Immunity.

[114] Zhang Y., Chandra V., Riquelme Sanchez E., Dutta P., Quesada P.R., Rakoski A., Zoltan M., Arora N., Baydogan S., Horne W. (2020). Interleukin-17-induced neutrophil extracellular traps mediate resistance to checkpoint blockade in pancreatic cancer. J. Exp. Med..

[115] Canè S., Barouni R.M., Fabbi M., Cuozzo J., Fracasso G., Adamo A., Ugel S., Trovato R., De Sanctis F., Giacca M. (2023). Neutralization of NET-associated human ARG1 enhances cancer immunotherapy. Sci. Transl. Med..

[116] Mousset A., Lecorgne E., Bourget I., Lopez P., Jenovai K., Cherfils-Vicini J., Dominici C., Rios G., Girard-Riboulleau C., Liu B. (2023). Neutrophil extracellular traps formed during chemotherapy confer treatment resistance via TGF-β activation. Cancer Cell.

[117] Mousset A., Bellone L., Gaggioli C., Albrengues J. (2024). NETscape or NEThance: Tailoring anti-cancer therapy. Trends Cancer.

[118] Maddalena M., Dimitrov J., Mehmood T., Terlizzi C., Esposito P.M.H., Franzese A., Pellegrino S., De Rosa V., Iommelli F., Del Vecchio S. (2025). Neutrophil extracellular traps as drivers of epithelial-mesenchymal transition in cancer cells. Front. Immunol..

[119] Lee J.H., Sánchez-Rivera F.J., He L., Basnet H., Chen F.X., Spina E., Li L., Torner C., Chan J.E., Yarlagadda D.V.K. (2024). TGF-β and RAS jointly unmask primed enhancers to drive metastasis. Cell.

[120] Cools-Lartigue J., Spicer J., McDonald B., Gowing S., Chow S., Giannias B., Bourdeau F., Kubes P., Ferri L. (2013). Neutrophil extracellular traps sequester circulating tumor cells and promote metastasis. J. Clin. Investig..

[121] Yang L., Liu Q., Zhang X., Liu X., Zhou B., Chen J., Huang D., Li J., Li H., Chen F. (2020). DNA of neutrophil extracellular traps promotes cancer metastasis via CCDC25. Nature.

[122] Xu S., Liu Y., Zhang T., Zheng J., Lin W., Cai J., Zou J., Chen Y., Xie Y., Chen Y. (2021). The Global, Regional, and National Burden and Trends of Breast Cancer From 1990 to 2019: Results From the Global Burden of Disease Study 2019. Front. Oncol..

[123] Senkus E., Kyriakides S., Ohno S., Penault-Llorca F., Poortmans P., Rutgers E., Zackrisson S., Cardoso F. (2015). Primary breast cancer: ESMO Clinical Practice Guidelines for diagnosis, treatment and follow-up. Ann. Oncol..

[124] de la Peña F.A., Novoa S.A., Gregori J.G., Cortijo L.G., Carrasco F.H., Martínez Martínez M.T., Estévez C.M., Stradella A., Losada M.J.V., Ciruelos E. (2026). SEOM-GEICAM-SOLTI clinical guidelines for early-stage breast cancer (UPDATE 2025). Clin. Transl. Oncol..

[125] Fumagalli C., Barberis M. (2021). Breast Cancer Heterogeneity. Diagnostics.

[126] Harbeck N., Penault-Llorca F., Cortes J., Gnant M., Houssami N., Poortmans P., Ruddy K., Tsang J., Cardoso F. (2019). Breast cancer. Nat. Rev. Dis. Primers.

[127] Wu G.Y., Xiao M.Z., Hao W.C., Yang Z.S., Liu X.R., Xu D.S., Peng Z.X., Zhang L.Y. (2025). Drug resistance in breast cancer: Mechanisms and strategies for management. Drug Resist. Updates.

[128] Rodríguez-Bejarano O.H., Parra-López C., Patarroyo M.A. (2024). A review concerning the breast cancer-related tumour microenvironment. Crit. Rev. Oncol. Hematol..

[129] Yuan J., Yang L., Li Z., Zhang H., Wang Q., Huang J., Wang B., Mohan C.D., Sethi G., Wang G. (2023). The role of the tumor microenvironment in endocrine therapy resistance in hormone receptor-positive breast cancer. Front. Endocrinol..

[130] Guo Z., Zhang H., Fu Y., Kuang J., Zhao B., Zhang L., Lin J., Lin S., Wu D., Xie G. (2023). Cancer-associated fibroblasts induce growth and radioresistance of breast cancer cells through paracrine IL-6. Cell Death Discov..

[131] Brogna M.R., Varone V., DelSesto M., Ferrara G. (2025). The role of CAFs in therapeutic resistance in triple negative breast cancer: An emerging challenge. Front. Mol. Biosci..

[132] Stavrou M., Constantinidou A. (2024). Tumor associated macrophages in breast cancer progression: Implications and clinical relevance. Front. Immunol..

[133] Vyas D., Laput G., Vyas A.K. (2014). Chemotherapy-enhanced inflammation may lead to the failure of therapy and metastasis. Onco Targets Ther..

[134] Moura T., Laranjeira P., Caramelo O., Gil A.M., Paiva A. (2025). Breast Cancer and Tumor Microenvironment: The Crucial Role of Immune Cells. Curr. Oncol..

[135] Correia B.F., Grosa D., Salvador R., Brites I., Martins T., Vitorino M., Sousa C.X., Cristóvão-Ferreira S., Braga S., Jacinto A. (2025). Neutrophils matter: New clinical insights on their role in the progression of metastatic breast cancer. Breast Cancer Res..

[136] Wen S., Feng T., Fan Y. (2025). Tumor-associated neutrophils in breast cancer: An angel or a devil?. Front. Immunol..

[137] Obeagu E.I., Ezeala C.C. (2025). Neutrophils as key regulators of tumor microenvironment in breast cancer: A focus on N1 and N2 polarization. Ann. Med. Surg..

[138] Papadopoulou S., Michou V., Tsiotsias A., Tzitiridou-Chatzopoulou M., Eskitzis P. (2026). Tumor-Associated Neutrophils and Desmoplastic Reaction in the Breast Cancer Tumor Microenvironment: A Comprehensive Review. Cancers.

[139] Norouzi F., Eini P., Tahmasebi S. (2025). The role of NETosis in breast cancer: Mechanistic insights and biomarker potential. Breast Cancer Res..

[140] Snoderly H.T., Boone B.A., Bennewitz M.F. (2019). Neutrophil extracellular traps in breast cancer and beyond: Current perspectives on NET stimuli, thrombosis and metastasis, and clinical utility for diagnosis and treatment. Breast Cancer Res..

[141] Zhao H., Liang Y., Sun C., Zhai Y., Li X., Jiang M., Yang R., Li X., Shu Q., Kai G. (2022). Dihydrotanshinone I Inhibits the Lung Metastasis of Breast Cancer by Suppressing Neutrophil Extracellular Traps Formation. Int. J. Mol. Sci..

[142] Xu X., Wang X., Zheng Z., Guo Y., He G., Wang Y., Fu S., Zheng C., Deng X. (2024). Neutrophil Extracellular Traps in Breast Cancer: Roles in Metastasis and Beyond. J. Cancer.

[143] Zhu D., Lu Y., Hu B., Pang Y., Liu B., Zhang M., Wang W., Wang Y. (2023). Highly-tumor-targeted PAD4 inhibitors with PBA modification inhibit tumors in vivo by specifically inhibiting the PAD4-H3cit-NETs pathway in neutrophils. Eur. J. Med. Chem..

[144] McDonald P.C., Dedhar S. (2022). New Perspectives on the Role of Integrin-Linked Kinase (ILK) Signaling in Cancer Metastasis. Cancers.

[145] Li N., Yin C., Tao J. (2025). Neutrophil extracellular traps in tumor metastasis: Mechanisms, and therapeutic implications. Discov. Oncol..

[146] Martins-Cardoso K., Almeida V.H., Bagri K.M., Rossi M.I.D., Mermelstein C.S., König S., Monteiro R.Q. (2020). Neutrophil Extracellular Traps (NETs) Promote Pro-Metastatic Phenotype in Human Breast Cancer Cells through Epithelial-Mesenchymal Transition. Cancers.

[147] Kong L., Hu S., Zhao Y., Huang Y., Xiang X., Yu Y., Mao X., Xie K., Zhu X., Xu P. (2025). Neutrophil extracellular traps induced by neoadjuvant chemotherapy of breast cancer promotes vascular endothelial damage. Breast Cancer Res..

[148] Shakerdi A.L., Finnegan E., Sheng Y.Y., Vidovic K., Logan J.M., Ward M.P., O’Toole S.A., Martin C., Selemidis S., Brooks D. (2025). Crosstalk Between Inflammasome Signalling and Epithelial-Mesenchymal Transition in Cancer and Benign Disease: Mechanistic Insights, Context-Dependence, and Therapeutic Opportunities. Cells.

[149] Zheng W., Marini W., Murakami K., Sotov V., Butler M., Gorrini C., Ohashi P.S., Reedijk M. (2024). Caspase-1-dependent spatiality in triple-negative breast cancer and response to immunotherapy. Nat. Commun..

[150] Mousset A., Albrengues J. (2024). Neutrophil extracellular traps modulate chemotherapy efficacy and its adverse side effects. Biol. Cell.

[151] Heiserman J.P., Akhurst R.J. (2025). Diverse Biological Processes Contribute to Transforming Growth Factor β-Mediated Cancer Drug Resistance. Cells.

[152] Cecerska-Heryć E., Jerzyk A., Goszka M., Polikowska A., Rachwalska J., Serwin N., Wojciuk B., Dołęgowska B. (2025). TGF-β Signaling in Cancer: Mechanisms of Progression and Therapeutic Targets. Int. J. Mol. Sci..

[153] Nakazawa D., Masuda S., Nishibata Y., Watanabe-Kusunoki K., Tomaru U., Ishizu A. (2025). Neutrophils and NETs in kidney disease. Nat. Rev. Nephrol..

[154] Xu H., Chen X., Lu Y., Sun N., Weisgerber K.E., Xu M., Bai R.Y. (2025). Neutrophil Dynamics in Response to Cancer Therapies. Cancers.

[155] Fan W., Fang Z., Weng Y., Zhan T., Huang K., Pan J., Zhan R. (2025). Iron enhances reactive oxygen species generation and initiates neutrophil extracellular traps formation on the endothelium to exacerbate stroke. J. Cell Commun. Signal..

[156] Espiritu A., O’Sullivan K.M. (2025). A Web of Challenges: The Therapeutic Struggle to Target NETs in Disease. Int. J. Mol. Sci..

[157] Hossain M.S., Karuniawati H., Jairoun A.A., Urbi Z., Ooi J., John A., Lim Y.C., Kibria K.M.K., Mohiuddin A.K.M., Ming L.C. (2022). Colorectal Cancer: A Review of Carcinogenesis, Global Epidemiology, Current Challenges, Risk Factors, Preventive and Treatment Strategies. Cancers.

[158] Cervantes A., Adam R., Roselló S., Arnold D., Normanno N., Taïeb J., Seligmann J., De Baere T., Osterlund P., Yoshino T. (2023). Metastatic colorectal cancer: ESMO Clinical Practice Guideline for diagnosis, treatment and follow-up. Ann. Oncol..

[159] Storli P.E., Dille-Amdam R.G., Skjerseth G.H., Gran M.V., Myklebust T., Grønbech J.E., Bringeland E.A. (2025). Synchronous metastases from colorectal cancer. Treatment and long-term survival compared to patients with metachronous metastases: A population-based study from Central Norway 2001–2015. Acta Oncol..

[160] Pericay C., Fernández Montes A., Alonso Orduña V., Macias Declara I., Asensio Martínez E., Rodríguez Salas N., Torres E., Cacho Lavín D., Rodríguez Alonso R.M., Falcó E. (2023). Real-World Outcomes in Patients with Metastatic Colorectal Cancer in Spain: The RWD-ACROSS Study. Cancers.

[161] Tie J., Cohen J.D., Lahouel K., Lo S.N., Wang Y., Kosmider S., Wong R., Shapiro J., Lee M., Harris S. (2022). Circulating Tumor DNA Analysis Guiding Adjuvant Therapy in Stage II Colon Cancer. N. Engl. J. Med..

[162] Grange R., Wagner M., Benzerdjeb N., Glehen O., Kepenekian V., Si-Mohamed S., Rousset P. (2026). Spectral CT imaging in colorectal cancer: Current applications, limitations, and future perspectives. Insights Imaging.

[163] Underwood P.W., Ruff S.M., Pawlik T.M. (2024). Update on Targeted Therapy and Immunotherapy for Metastatic Colorectal Cancer. Cells.

[164] Oh J.M., Kim S., Tsung C., Kent E., Jain A., Ruff S.M., Zhang H. (2025). Comprehensive review of the resistance mechanisms of colorectal cancer classified by therapy type. Front. Immunol..

[165] Källberg J., Harrison A., March V., Bērziņa S., Nemazanyy I., Kepp O., Kroemer G., Mouillet-Richard S., Laurent-Puig P., Taly V. (2023). Intratumor heterogeneity and cell secretome promote chemotherapy resistance and progression of colorectal cancer. Cell Death Dis..

[166] Langerud J., Eilertsen I.A., Moosavi S.H., Klokkerud S.M.K., Reims H.M., Backe I.F., Hektoen M., Sjo O.H., Jeanmougin M., Tejpar S. (2024). Multiregional transcriptomics identifies congruent consensus subtypes with prognostic value beyond tumor heterogeneity of colorectal cancer. Nat. Commun..

[167] Braumüller H., Mauerer B., Andris J., Berlin C., Wieder T., Kesselring R. (2022). The Cytokine Network in Colorectal Cancer: Implications for New Treatment Strategies. Cells.

[168] Morris V.K., Kennedy E.B., Baxter N.N., Benson A.B., Cercek A., Cho M., Ciombor K.K., Cremolini C., Davis A., Deming D.A. (2023). Treatment of Metastatic Colorectal Cancer: ASCO Guideline. J. Clin. Oncol..

[169] Zhou R.W., Harpaz N., Itzkowitz S.H., Parsons R.E. (2023). Molecular mechanisms in colitis-associated colorectal cancer. Oncogenesis.

[170] Han J., Liang W., Li K. (2026). Unveiling the tumor microenvironment in colorectal cancer therapeutic resistance. Front. Cell Dev. Biol..

[171] Brandaleone L., Dal Buono A., Gabbiadini R., Marcozzi G., Polverini D., Carvello M., Spinelli A., Hassan C., Repici A., Bezzio C. (2024). Hereditary Colorectal Cancer Syndromes and Inflammatory Bowel Diseases: Risk Management and Surveillance Strategies. Cancers.

[172] Liu W., Kuang T., Liu L., Deng W. (2024). The role of innate immune cells in the colorectal cancer tumor microenvironment and advances in anti-tumor therapy research. Front. Immunol..

[173] Masui H., Kawada K., Obama K. (2024). Neutrophil and Colorectal Cancer. Int. J. Mol. Sci..

[174] Xu Z.X., Qu F.Y., Zhang Z., Luan W.Y., Lin S.X., Miao Y.D. (2025). Exploring the role of neutrophil extracellular traps in colorectal cancer: Insights from single-cell sequencing. World J. Gastrointest. Oncol..

[175] Khan U., Chowdhury S., Billah M.M., Islam K.M.D., Thorlacius H., Rahman M. (2021). Neutrophil Extracellular Traps in Colorectal Cancer Progression and Metastasis. Int. J. Mol. Sci..

[176] Zheng W., Wu J., Peng Y., Sun J., Cheng P., Huang Q. (2022). Tumor-Associated Neutrophils in Colorectal Cancer Development, Progression and Immunotherapy. Cancers.

[177] Wang X., He S., Gong X., Lei S., Zhang Q., Xiong J., Liu Y. (2025). Neutrophils in colorectal cancer: Mechanisms, prognostic value, and therapeutic implications. Front. Immunol..

[178] Schimek V., Strasser K., Beer A., Göber S., Walterskirchen N., Brostjan C., Müller C., Bachleitner-Hofmann T., Bergmann M., Dolznig H. (2022). Tumour cell apoptosis modulates the colorectal cancer immune microenvironment via interleukin-8-dependent neutrophil recruitment. Cell Death Dis..

[179] Verbeke H., Struyf S., Laureys G., Van Damme J. (2011). The expression and role of CXC chemokines in colorectal cancer. Cytokine Growth Factor Rev..

[180] Sun R., Xiong Y., Liu H., Gao C., Su L., Weng J., Yuan X., Zhang D., Feng J. (2020). Tumor-associated neutrophils suppress antitumor immunity of NK cells through the PD-L1/PD-1 axis. Transl. Oncol..

[181] Yajuk O., Baron M., Toker S., Zelter T., Fainsod-Levi T., Granot Z. (2021). The PD-L1/PD-1 Axis Blocks Neutrophil Cytotoxicity in Cancer. Cells.

[182] Ozel I., Sha G., Będzińska A., Pylaeva E., Naumova Y., Thiel I., Antczak J., Squire A., Gunzer M., Zelinskyy G. (2025). Neutrophil-specific targeting of STAT3 impairs tumor progression via the expansion of cytotoxic CD8^+^ T cells. Signal Transduct. Target. Ther..

[183] Wang B., Su X., Zhang B., Pan S. (2023). GSK484, an inhibitor of peptidyl arginine deiminase 4, increases the radiosensitivity of colorectal cancer and inhibits neutrophil extracellular traps. J. Gene Med..

[184] Zhu D., Lu Y., Wang Y., Wang Y. (2022). PAD4 and Its Inhibitors in Cancer Progression and Prognosis. Pharmaceutics.

[185] Li D., Shao J., Cao B., Zhao R., Li H., Gao W., Chen P., Jin L., Cao L., Ji S. (2022). The Significance of Neutrophil Extracellular Traps in Colorectal Cancer and Beyond: From Bench to Bedside. Front. Oncol..

[186] Wang Y., Yang K., Li J., Wang C., Li P., Du L. (2025). Neutrophil extracellular traps in cancer: From mechanisms to treatments. Clin. Transl. Med..

[187] Nors J., Iversen L.H., Erichsen R., Gotschalck K.A., Andersen C.L. (2024). Incidence of Recurrence and Time to Recurrence in Stage I to III Colorectal Cancer: A Nationwide Danish Cohort Study. JAMA Oncol..

[188] Bezu L., Akçal Öksüz D., Bell M., Buggy D., Diaz-Cambronero O., Enlund M., Forget P., Gupta A., Hollmann M.W., Ionescu D. (2024). Perioperative Immunosuppressive Factors during Cancer Surgery: An Updated Review. Cancers.

[189] Carroll G.M., Burns G.L., Petit J.A., Walker M.M., Mathe A., Smith S.R., Keely S., Pockney P.G. (2020). Does postoperative inflammation or sepsis generate neutrophil extracellular traps that influence colorectal cancer progression? A systematic review. Surg. Open Sci..

[190] Teijeira A., Garasa S., Ochoa M.C., Villalba M., Olivera I., Cirella A., Eguren-Santamaria I., Berraondo P., Schalper K.A., de Andrea C.E. (2021). IL8, Neutrophils, and NETs in a Collusion against Cancer Immunity and Immunotherapy. Clin. Cancer Res..

[191] Xu Z., Feng H., Feng W., Zhang Y., Huo J., Xu Z., Chen F., Gao H., Lv Z., Liu W. (2025). Agonist signaling drives neutrophil subpopulations to promote/inhibit colorectal cancer liver metastasis. Nat. Commun..

[192] Zhang Y., Song J., Zhang Y., Li T., Peng J., Zhou H., Zong Z. (2022). Emerging Role of Neutrophil Extracellular Traps in Gastrointestinal Tumors: A Narrative Review. Int. J. Mol. Sci..

[193] Alfaro C., Teijeira A., Oñate C., Pérez G., Sanmamed M.F., Andueza M.P., Alignani D., Labiano S., Azpilikueta A., Rodriguez-Paulete A. (2016). Tumor-Produced Interleukin-8 Attracts Human Myeloid-Derived Suppressor Cells and Elicits Extrusion of Neutrophil Extracellular Traps (NETs). Clin. Cancer Res..

[194] Okamoto M., Mizuno R., Kawada K., Itatani Y., Kiyasu Y., Hanada K., Hirata W., Nishikawa Y., Masui H., Sugimoto N. (2023). Neutrophil Extracellular Traps Promote Metastases of Colorectal Cancers through Activation of ERK Signaling by Releasing Neutrophil Elastase. Int. J. Mol. Sci..

[195] Ying Y., Wang H., Wang Y., Huang J., Wu Z., Qiu B., Chen H., Long M., Mo K., Cui C. (2025). PPIF^+^ neutrophils promote mtROS driven NETosis mediated progression of colorectal cancer. J. Transl. Med..

[196] Rayes R.F., Vourtzoumis P., Bou Rjeily M., Seth R., Bourdeau F., Giannias B., Berube J., Huang Y.H., Rousseau S., Camilleri-Broet S. (2020). Neutrophil Extracellular Trap-Associated CEACAM1 as a Putative Therapeutic Target to Prevent Metastatic Progression of Colon Carcinoma. J. Immunol..

[197] Tohme S., Yazdani H.O., Al-Khafaji A.B., Chidi A.P., Loughran P., Mowen K., Wang Y., Simmons R.L., Huang H., Tsung A. (2016). Neutrophil Extracellular Traps Promote the Development and Progression of Liver Metastases after Surgical Stress. Cancer Res..

[198] Li Y., Wu S., Zhao Y., Dinh T., Jiang D., Selfridge J.E., Myers G., Wang Y., Zhao X., Tomchuck S. (2024). Neutrophil extracellular traps induced by chemotherapy inhibit tumor growth in murine models of colorectal cancer. J. Clin. Investig..

[199] SenGupta S., Hein L.E., Parent C.A. (2021). The Recruitment of Neutrophils to the Tumor Microenvironment Is Regulated by Multiple Mediators. Front. Immunol..

[200] Azzouz D., Palaniyar N. (2024). How Do ROS Induce NETosis? Oxidative DNA Damage, DNA Repair, and Chromatin Decondensation. Biomolecules.

[201] Park W., Chawla A., O’Reilly E.M. (2021). Pancreatic Cancer: A Review. JAMA.

[202] Blackford A.L., Canto M.I., Dbouk M., Hruban R.H., Katona B.W., Chak A., Brand R.E., Syngal S., Farrell J., Kastrinos F. (2024). Pancreatic Cancer Surveillance and Survival of High-Risk Individuals. JAMA Oncol..

[203] Bugazia D., Al-Najjar E., Esmail A., Abdelrahim S., Abboud K., Abdelrahim A., Umoru G., Rayyan H.A., Abudayyeh A., Al Moustafa A.E. (2024). Pancreatic ductal adenocarcinoma: The latest on diagnosis, molecular profiling, and systemic treatments. Front. Oncol..

[204] Taherian M., Wang H., Wang H. (2022). Pancreatic Ductal Adenocarcinoma: Molecular Pathology and Predictive Biomarkers. Cells.

[205] Zeng S., Pöttler M., Lan B., Grützmann R., Pilarsky C., Yang H. (2019). Chemoresistance in Pancreatic Cancer. Int. J. Mol. Sci..

[206] Conroy T., Ducreux M. (2025). ESMO Clinical Practice Guideline Express Update on the management of metastatic pancreatic cancer. ESMO Open.

[207] Deng D., Patel R., Chiang C.Y., Hou P. (2022). Role of the Tumor Microenvironment in Regulating Pancreatic Cancer Therapy Resistance. Cells.

[208] Lin Z., Adeniran E.A., Cai Y., Qureshi T.A., Li D., Gong J., Li J., Pandol S.J., Jiang Y. (2025). Early Detection of Pancreatic Cancer: Current Advances and Future Opportunities. Biomedicines.

[209] Galindo-Vega A., Maldonado-Lagunas V., Mitre-Aguilar I.B., Melendez-Zajgla J. (2023). Tumor Microenvironment Role in Pancreatic Cancer Stem Cells. Cells.

[210] Stoop T.F., Javed A.A., Oba A., Koerkamp B.G., Seufferlein T., Wilmink J.W., Besselink M.G. (2025). Pancreatic cancer. Lancet.

[211] Tempero M.A., Pelzer U., O’Reilly E.M., Winter J., Oh D.Y., Li C.P., Tortora G., Chang H.M., Lopez C.D., Bekaii-Saab T. (2023). Adjuvant nab-Paclitaxel + Gemcitabine in Resected Pancreatic Ductal Adenocarcinoma: Results From a Randomized, Open-Label, Phase III Trial. J. Clin. Oncol..

[212] Zhu T., Yang Q., Qian X., Wu X., Fang J., Lin Y., Feng Y., Gao J., Xia Q. (2025). GPRC5A/CXCL8/NLRP3-mediated neutrophil extracellular traps drive gemcitabine-nab-paclitaxel resistance in pancreatic adenocarcinoma. Cancer Biol. Med..

[213] Nywening T.M., Belt B.A., Cullinan D.R., Panni R.Z., Han B.J., Sanford D.E., Jacobs R.C., Ye J., Patel A.A., Gillanders W.E. (2018). Targeting both tumour-associated CXCR2^+^ neutrophils and CCR2^+^ macrophages disrupts myeloid recruitment and improves chemotherapeutic responses in pancreatic ductal adenocarcinoma. Gut.

[214] Sturgeon R., Goel P., Singh R.K. (2023). Tumor-associated neutrophils in pancreatic cancer progression and metastasis. Am. J. Cancer Res..

[215] Antuamwine B.B., Bosnjakovic R., Hofmann-Vega F., Wang X., Theodosiou T., Iliopoulos I., Brandau S. (2023). N1 versus N2 and PMN-MDSC: A critical appraisal of current concepts on tumor-associated neutrophils and new directions for human oncology. Immunol. Rev..

[216] Jin W., Xu H.X., Zhang S.R., Li H., Wang W.Q., Gao H.L., Wu C.T., Xu J.Z., Qi Z.H., Li S. (2019). Tumor-Infiltrating NETs Predict Postsurgical Survival in Patients with Pancreatic Ductal Adenocarcinoma. Ann. Surg. Oncol..

[217] Qu M., Zhu C., Sun C., Zhu S., Zhang H., Miao C., Zhou D. (2025). Neutrophil Extracellular Traps Promote Pancreatic Cancer Progression via the STING Pathway. Gastroenterol. Res. Pract..

[218] Chen X., Ma H., Mo S., Yu S., Lu Z., Chen J. (2022). Intratumoral neutrophil extracellular traps are associated with unfavorable clinical outcomes and immunogenic context in pancreatic ductal adenocarcinoma. Front. Immunol..

[219] Fu Y., Tao J., Gu Y., Liu Y., Qiu J., Su D., Wang R., Luo W., Liu T., Zhang F. (2024). Multiomics integration reveals NETosis heterogeneity and TLR2 as a prognostic biomarker in pancreatic cancer. NPJ Precis. Oncol..

[220] Hackner D., Merkel S., Weiß A., Krautz C., Weber G.F., Grützmann R., Brunner M. (2024). Neutrophil-to-Lymphocyte Ratio and Prognostic Nutritional Index Are Predictors for Overall Survival after Primary Pancreatic Resection of Pancreatic Ductal Adenocarcinoma: A Single Centre Evaluation. Cancers.

[221] Jin W., Yin H., Li H., Yu X.J., Xu H.X., Liu L. (2021). Neutrophil extracellular DNA traps promote pancreatic cancer cells migration and invasion by activating EGFR/ERK pathway. J. Cell. Mol. Med..

[222] McDonald P.C., Topham J.T., Awrey S., Tavakoli H., Carroll R., Brown W.S., Gerbec Z.J., Kalloger S.E., Karasinska J.M., Tang P. (2025). Neutrophil extracellular trap gene expression signatures identify prognostic and targetable signaling axes for inhibiting pancreatic tumour metastasis. Commun. Biol..

[223] Mitsis M., Drosou P., Tatsis V., Markopoulos G.S. (2022). Neutrophil Extracellular Traps and Pancreatic Cancer Development: A Vicious Cycle. Cancers.

[224] Nogi S., Kagawa S., Taniguchi A., Yagi T., Kanaya N., Kakiuchi Y., Yasui K., Fuji T., Kono Y., Kikuchi S. (2025). Gemcitabine-induced neutrophil extracellular traps via interleukin-8-CXCR1/2 pathway promote chemoresistance in pancreatic cancer. Br. J. Cancer.

[225] Han Z.J., Li Y.B., Yang L.X., Cheng H.J., Liu X., Chen H. (2021). Roles of the CXCL8-CXCR1/2 Axis in the Tumor Microenvironment and Immunotherapy. Molecules.

[226] Amo-Aparicio J., Dominguez A., Atif S.M., Dinarello A., Azam T., Alula K.M., Piper M., Lieu C.H., Lentz R.W., Leal A.D. (2023). Pancreatic Ductal Adenocarcinoma Cells Regulate NLRP3 Activation to Generate a Tolerogenic Microenvironment. Cancer Res. Commun..

